# Neural Correlates of Rhythm in Post-Stroke Aphasia

**DOI:** 10.1162/nol.a.9

**Published:** 2025-08-14

**Authors:** Anna V. Kasdan, Marianne Casilio, Katherine Bryan, Nori Jacoby, Noah R. Fram, Lily Walljasper, Deborah F. Levy, Michael de Riesthal, Reyna L. Gordon, Stephen M. Wilson

**Affiliations:** Department of Hearing and Speech Sciences, Vanderbilt University Medical Center, Nashville, TN, USA; Max Planck Institute for Empirical Aesthetics, Frankfurt, Germany; Department of Otolaryngology—Head & Neck Surgery, Vanderbilt University Medical Center, Nashville, TN, USA; Department of Neurological Surgery, University of California, San Francisco, San Francisco, CA, USA; School of Health and Rehabilitation Sciences, University of Queensland, Brisbane, QLD, Australia

**Keywords:** aphasia, lesion-symptom mapping, music, rhythm, tapping

## Abstract

Individuals with post-stroke aphasia have long been observed to show relatively preserved musical and rhythm abilities in the presence of varied, and often profound, language impairments. Accordingly, speech-language pathologists frequently use rhythm-based strategies (e.g., tapping) to facilitate speech output in people with aphasia. However, there is little empirical work to support the clinical practice of using rhythm techniques. In this study, we investigated the neural bases of rhythm in aphasia by combining thorough behavioral rhythm assessments with structural brain imaging. Individuals with chronic, post-stroke aphasia (*n* = 33) and a matched neurotypical control group (*n* = 29) completed a rigorous battery of rhythm production and perception tasks. We found marked individual variability within the aphasia group, with about one third of individuals showing impaired rhythm processing, while the remaining two thirds performed within the control range. Using lesion-symptom mapping, we found that individual variability in tapping performance was associated with damage to a left temporoparietal area, extending into white matter specifically in the arcuate fasciculus. That is, individuals who struggled with tapping tended to have damage to this region. Tapping was also associated with language production scores, but not motor speech, in the aphasia group. These findings, which systematically link rhythm, language, and the brain, have the potential to be translated into clinical practice for understanding which patients may benefit the most from rhythm-based treatments. Our study in a population with focal brain injury complements evolutionary work highlighting the importance of the left temporoparietal region and underlying white matter for beat synchronization.

## INTRODUCTION

For an accessible, aphasia-friendly (Kasdan et al., 2025) version of this paper, please see Tapping to a Beat in Aphasia in the Supplementary Materials, available at https://doi.org/10.1162/nol.a.9.

For centuries, clinicians and researchers have observed that individuals with aphasia can show spared musical abilities in the presence of varied and profound language impairments. These early reports (Gerstman, 1964; Symonds, 1953) fueled the use of rhythm techniques in clinic, which are used frequently by speech-language pathologists both as impromptu tools (e.g., tapping to a beat, pacing) to facilitate speech output and as part of established protocols, including speech entrainment (Fridriksson et al., 2012) and, most notably, melodic intonation therapy (MIT; Albert et al., 1973; Sparks et al., 1974; Sparks & Holland, 1976). MIT has been recognized as one of the most formalized therapies for aphasia (Benson et al., 1994). While these tools and therapies have many active ingredients, rhythm is a common factor among many of them. For example, the unison singing component of MIT involves anticipation of temporally predictable information and can provide a speech rhythm “scaffold” conveyed by the speech envelope (Poeppel & Assaneo, 2020); some work suggests the rhythmic dimension of MIT may be a driving factor in its efficacy (Boucher et al., 2001; Stahl et al., 2011). Speech entrainment and script training similarly use rhythmic elements such as exaggerated prosody to improve speech fluency (Cherney & Halper, 2008; Cherney et al., 2008; Feenaughty et al., 2021; Fridriksson et al., 2012).

Despite the frequent clinical use of rhythm techniques, there is little empirical research on rhythm processing in aphasia at both a neural and a behavioral level. Neural information is crucial to investigate given that lesion location is the most important predictor of recovery from aphasia (Wilson et al., 2023). Given that rhythm seems to be represented bilaterally in the brain (Kasdan et al., 2022) and that post-stroke aphasia is almost always caused by lesions to the left hemisphere (Berthier, 2005; Pedersen et al., 1995), a key, open question is: Do unilateral, left hemisphere lesions lead to impairments in rhythm production?

There are only two studies up to this point that have systematically investigated behavioral rhythm abilities in post-stroke aphasia. One experimental study (Zipse et al., 2014) found that participants with aphasia performed below the level of controls on a majority of rhythm perception and production measures; however, there was also significant individual variability. The second of these studies found that individuals with aphasia performed comparably to controls on rhythm discrimination tasks (Stefaniak et al., 2021). Their main finding—that participants with better beat-based rhythm perception had greater speech output fluency—corroborates the modest amount of treatment literature on using rhythm and timing elements to increase fluency in aphasia, and also aligns with work suggesting that people with aphasia may benefit from synchronizing speech with an external rhythmic stimulus (Stahl et al., 2011).

Rhythm-based therapies for aphasia, including MIT and its related variants (Baker, 2000), assume that rhythm abilities are intact in this population. Both MIT and speech entrainment are thought to be best suited for a relatively select group of patients who fit the following criteria: (1) nonfluent/Broca’s aphasia with limited verbal output, (2) reasonably preserved auditory comprehension, (3) poor repetition, and (4) little to no damage to posterior language areas (Benson et al., 1994; Fridriksson et al., 2012, 2015; Naeser & Helm-Estabrooks, 1985; Sparks et al., 1974). Mechanistically, it is thought that the rhythmic left-hand tapping and melodic intoning aspects of MIT engage a right-hemisphere network homotopic to the left-hemisphere language network (Berlin, 1976; Goldfarb & Bader, 1979; Sparks et al., 1974). These hypotheses—first outlined in the 1970s before the advent of neuroimaging—have advanced little since that time.

A recent survey study indicated that clinicians select to use MIT with severe global and Wernicke’s aphasia more so than other subgroups (Hinckley & Sanchez, 2023). This strays from the original recommendations outlined by the founders—in that people with global and Wernicke’s aphasias often have impaired auditory comprehension and large lesions spanning left posterior temporal regions (Wernicke, 1874; Wilson et al., 2023) and may not be good responders to MIT (Benson et al., 1994)—and is likely part of the reason why reports on the efficacy of MIT find it variable (van der Meulen et al., 2012). Early on, it was even noted that MIT would “probably not transcend” the “limitation” of language therapy for individuals with global aphasia (Sparks et al., 1974). Neuroimaging work suggests that MIT results in recovery through spared right hemisphere regions (Schlaug et al., 2008, 2009; Wan et al., 2014; Zipse et al., 2012), particularly a right sensorimotor network that is engaged through tapping; this body of work has concluded that MIT is suited for patients with large left hemisphere lesions who must instead rely on regions in the right for language recovery (Schlaug et al., 2010). This work has included individuals who have lesions spanning the entire left middle cerebral artery territory (Zipse et al., 2012), again at odds with ideas from the founders about optimal patients (Albert et al., 1973; Benson et al., 1994; Naeser & Helm-Estabrooks, 1985). In general, there is equivocal evidence to support reorganization of language to the right hemisphere (Wilson & Schneck, 2021) and indeed some PET/SPECT studies in patients who received MIT found activations in left prefrontal or perilesional areas (Belin et al., 1996; Martzoukou et al., 2021).

At present, the literature lacks clarity on how individuals with post-stroke aphasia perform on rhythm tasks (i.e., which individuals excel or struggle) and which brain areas support that performance; this lack of clarity stems in part from the disconnect between who rhythm-based treatments were originally developed for and who they are being used with now, both in neuroimaging studies and in clinical practice. It is difficult to make inferences about which brain regions are important for rhythm based on the small sample sizes, varied methods, and lack of specificity about lesion location in prior studies (e.g., Schlaug et al., 2009).

Given the need for a more comprehensive understanding on rhythm and the brain in chronic, post-stroke aphasia, we asked whether rhythm skills were associated with specific patterns of brain damage. Such work is needed to answer future, practical clinical questions aligned with precision medicine approaches (Collins & Varmus, 2015; Denny & Collins, 2021) such as: Who are the best candidates for rhythm-based interventions?

In the current study, we characterized rhythm abilities, and their relationship to lesion location and language profiles, in a cohort of participants twice as large as previous studies on this topic (Stahl et al., 2011; Stefaniak et al., 2021; Zipse et al., 2014). We assessed rhythm abilities in-depth using a carefully selected battery of beat-based rhythm tasks (Bégel et al., 2018; Dalla Bella et al., 2017; Iversen & Patel, 2008; Niarchou et al., 2022; Repp, 2005) that were adapted for individuals with aphasia. The present work employs both beat-based rhythm perception and production tasks; however, we focus mostly on rhythm production (i.e., tapping) tasks, which provide more direct links with techniques used in clinical practice.

## MATERIALS AND METHODS

### Participants

Thirty-three individuals with chronic, post-stroke aphasia and 29 neurotypical controls, matched on age and education level, successfully completed the research protocol. Participants in both groups were included if they were fluent in English and aged 18–90 years old; participants were excluded if they had a comorbid major neurological or psychiatric condition. We recruited individuals with aphasia who had aphasia due to ischemic or hemorrhagic stroke confined mostly to left hemisphere supratentorial regions (Wilson et al., 2023) and were in the chronic stage of recovery (approximately 1 yr or more post-stroke). Recruitment took place over a 1.5 year period. We recruited all individuals who met these criteria, regardless of musical background.

An additional three patients with aphasia were recruited but were not included in analyses due to either inability to achieve task set due to suspected interfering neurological/audiological problems at the time of testing (*n* = 1) or brain injuries with significant involvement of right hemisphere regions (*n* = 2). Demographic information is provided in Table 1.

**Table 1.** Participant demographics

	Aphasia (*n* = 33)	Controls (*n* = 29)
Age (yr)	58.4 ± 15.9	62.0 ± 16.6
Sex	9 F, 24 M	15 F, 14 M
Handedness	27 R; 3 L; 3 Am	25 R; 3 L; 1 Am
Education (yr)	15.1 ± 2.9	15.9 ± 1.7
Race	27 W; 5 B; 1 A	28 W; 1 B
Time post-stroke (mon)	57.1 ± 52.7 (range: 11–258)	NA
Stroke type	28 Isch, 5 Hem	NA
Lesion size (cm^3^)	121.3 ± 126.3 (range: 2.1–434.5)	NA

*Notes*. Mean ± standard deviation are presented except where noted. F = female; M = male; R = right-handed; L = left-handed; Am = ambidextrous; W = White; B = Black or African American; A = Asian; Isch = ischemic; Hem = hemorrhagic.

Participants with aphasia were recruited through three main avenues: (1) the Vanderbilt Language Neuroscience Lab database, where several individuals had participated in previous research studies in the lab; (2) the Aphasia Group of Middle Tennessee, a community aphasia group housed within the Pi Beta Phi Rehabilitation Institute (PBPRI) at Vanderbilt University Medical Center (VUMC; Levy et al., 2022), and (3) referrals from clinicians at PBPRI. Controls were recruited from the community through fliers and word-of-mouth.

The study was approved by Vanderbilt University’s Institutional Review Board (IRB). Neurotypical controls were provided with a standard language consent form, while individuals with aphasia were presented with an IRB-approved aphasia-friendly consent form (see Supplementary Materials) to make the content accessible. Participants were compensated in gift card format for their time.

#### Rhythm task battery

Participants in both groups completed three rhythm tasks—two rhythm production tasks (tapping to a metronome and tapping to the beat of music) and one beat-based rhythm perception task.

##### Rhythm production tasks: Isochronous tapping.

The ability to synchronize with a beat was assessed using a metronome (i.e., isochronous) tapping task. Participants tapped to three metronome sequences with interonset intervals (IOIs) of 600 ms, 450 ms, and 750 ms, in that order (Dalla Bella et al., 2017). Each sequence had 60 piano tones (note frequency: 165 Hz, or E3; note length: 125 ms). The beginning and end of each metronome sequence was marked with signal sounds (2 pure tones of 500 Hz and 100 ms duration with an IOI of 300 ms); participants were told to start tapping when the metronome started and to stop tapping when it ended. As many individuals with aphasia experience motor weakness on their right side, we instructed all participants to tap with their left hand for consistency. Only one neurotypical control tapped with their right hand, due to left-sided hand weakness unrelated to neurological problems.

The experiment was programmed in MATLAB and used a similar apparatus to prior tapping experiments (Jacoby & McDermott, 2017); this setup was the same for the music tapping task. Briefly, the setup consisted of a tapping sensor box constructed by the experimenters, a Focusrite Scarlett 2i2 USB sound card, a laptop, and Bose QuietComfort 45 Headphones. This setup allowed for simultaneous stimulus presentation and recording of participant taps. A sound check was completed prior to the start of the experiment to ensure a comfortable listening level for each participant, including the nine participants with aphasia and six controls who either self-reported hearing loss or were hearing aid users.

Prior to the main experiment, all participants completed a practice phase in Keynote slides, without headphones, which included the same three metronome tempi (but with sequences of only 15 piano tones). Various levels of scaffolding (e.g., tapping with participants) were employed to ensure that participants, particularly those with aphasia, understood the task instructions. Practice sequences were again completed in MATLAB.

##### Rhythm production task: Music tapping.

In this task, participants tapped along to the beat of music. The design and stimuli for this task were largely similar to previous work, which we have summarized below (Anglada-Tort et al., 2022; Niarchou et al., 2022).

Participants tapped to 12 trials—six unique musical excepts, each repeated twice, in a randomized order such that the same excerpt could not appear twice in a row. Each trial was marked with metronome signal sounds as described above.

Music clips were 30 seconds each and were selected from the MIREX 2006 Audio Beat Tracking database which also provides time series annotations of the beat locations from each of 40 listeners who tapped along to the music (McKinney et al., 2007). From the annotations, we extracted the true “beat locations” using kernel density estimation (kernel width = 20 ms); the peaks of the probability density function (i.e., the true beat locations) were identified using the findpeaks function in MATLAB with previously recommended parameters (Niarchou et al., 2022), with some slight modifications depending on the music clip. Details of the music excerpts are in Table S1 in Supplementary Data, Figures, and Tables.

To help participants find the beat of the music and eliminate any ambiguity related to double timing or half timing, a metronome (frequency: 1000 Hz; duration: 50 ms) marking the aforementioned beat locations was added for the first 11 seconds of each music clip (Niarchou et al., 2022). The metronome then disappeared and participants were instructed to keep tapping to the beat of the music as if the metronome were there.

The experimental setup and apparatus were identical to the isochronous tapping task and participants completed a similar practice phase with various levels of scaffolding plus two practice trials in MATLAB for signal quality checks. Together, the two rhythm production tasks took about 25 minutes to complete.

##### Rhythm perception task.

We used the Beat Alignment Test (BAT) as a measure of beat-based rhythm perception. In this task, participants heard a clip of instrumental music; all clips were taken from the original BAT (Iversen & Patel, 2008). About halfway through each clip, metronome beeps appeared that either aligned with the beat of the music or did not align (phase shifted ±30%). After the music clip was over, participants indicated via a button press on a keypad whether they thought the beeps aligned or did not align with the beat of the music. Participants were told to just listen to the music and do their best not to tap or otherwise move to the music. There were four practice trials prior to the start of the experiment.

We included 18 scored trials—nine on-beat and nine off-beat. Stimuli were counterbalanced across participants such that each participant received a unique ordering, with the constraints that (a) the same musical clip was never presented twice in a row and (b) the same condition was never presented three times in a row. Additionally, there were three “filler” off-beat trials that occurred in a fixed position for everyone to preclude any learning effects related to the equal number of scored on/off-beat trials. The “filler” stimuli were tempo-shifted rather than phase-shifted and were not scored. The task took approximately 10 minutes to complete.

#### Musical experience survey

All participants completed a short musical experience survey (MES) which consisted of four questions about musical training and one question about dance training. Questions were modified from the Ollen Musical Sophistication Index (Ollen, 2006). Participants with aphasia were presented with an aphasia-friendly version of the Musical Experience Survey.

Neurotypical controls also completed the Goldsmiths Musical Sophistication Index (GMSI; Müllensiefen et al., 2014), which additionally included one question from the Goldsmiths Dance Sophistication Index (Rose et al., 2022). Participants with aphasia did not complete this survey due to its length and complexity.

Questions for the shortened MES corresponded approximately to questions on the GMSI and were scored according to that ordinal scale (1–7); participants received a score of 1–7 for each of the five questions on the MES. The first four questions, which were about music training, were summed for a MES Total score, in line with how GMSI subscores are computed. More details on MES scoring procedures are provided in Supplementary Data, Figures, and Tables.

In controls, our MES correlated highly with the Musical Training subscale of the GMSI (*r* = 0.80, *p* < 0.001), suggesting our MES is an appropriate measure of our construct of interest—formal musical training. Because of this, all subsequent analyses involving musical experience use the MES Total measure.

#### Speech/language evaluation

The Quick Aphasia Battery (QAB) is a reliable and multidimensional assessment of language function that takes approximately 20 minutes to administer (Wilson et al., 2018, 2023). The QAB consists of eight subtests from which several summary measures of language function are derived: a QAB overall score, single word comprehension, sentence comprehension, word finding, grammatical construction, speech motor programming (i.e., apraxia of speech [AOS]), repetition, and reading. We also examined speech motor execution (i.e., dysarthria), which, unlike the other individual summary scores, does not contribute to the QAB overall score.

For individuals who were participating in concurrent research studies in the lab, and thus their QAB assessment was being used for multiple projects (*n* = 7), we administered the extended version of the QAB (Casilio et al., 2024; Wilson et al., 2023). This longer version includes extra items for the word and sentence comprehension sections but was otherwise scored the same as the main version. Sessions were scored offline according to established protocols (Wilson et al., 2023).

#### Motor assessments

Participants with aphasia completed two motor assessments. The first was an assessment of gross motor function—the motor arm subtest of the NIH Stroke Scale (NIHSS; National Institute of Neurological Disorders and Stroke, 1998). In this assessment, participants must hold out their arm at 90° for 10 seconds and are scored on the amount of drift during that time. This is first completed on the left side and then repeated on the right side.

The second motor test was the Apraxia Screen of TULIA (AST)—a screening test for upper limb apraxia (Vanbellingen et al., 2011). Limb apraxia is an impairment in planning and executing skilled actions (e.g., motor substitutions, wrong postures) due to neurological dysfunction (Buxbaum & Randerath, 2018; Ochipa & Rothi, 2000). The AST consists of 12 items (7 imitation items and 5 pantomime items) extracted from a more comprehensive test for upper limb apraxia (Vanbellingen et al., 2010). The AST is quick to administer, reliable, and sensitive for detecting the presence of limb apraxia. All participants with aphasia completed the AST on their left side and participants without right-sided hemiparesis (*n* = 22) also completed the 12 AST items on their right side. Further details on items and test administration are described in Supplementary Data, Figure, and Tables.

Each item on the AST was scored on a dichotomous scale (0 = *fail*; 1 = *pass*). Three individuals had comprehension impairments that the research team deemed were significant enough to interfere with successful completion of the self-initiation items; these individuals were instead scored along the AST revised guidelines with a total of seven items. We calculated a scaled score for each individual based on the total number of items received.

### Procedure

For the most part, participants with aphasia completed the above tasks in a fixed order: isochronous tapping, music tapping, BAT, MES, QAB, NIHSS, AST. In some cases, administering tasks in this fixed order was not possible, such as in the few cases when sessions spanned more than 1 day (*n* = 5) and in cases where the experimenter needed to build rapport with the participant, in which case the QAB, with the connected speech portion at the beginning, was administered first. Neurotypical controls completed the tasks in a similar fixed order: isochronous tapping, music tapping, BAT, MESs.

Language and motor assessments (QAB, NIHSS, AST) for individuals with aphasia were scored offline using video and audio recordings. All assessments were double scored and then discussed in consensus meetings by the first author and a certified speech-language pathologist. Outstanding questions were resolved in additional consensus meetings with other certified speech-language pathologists or aphasia researchers (authors MC, KB, LW, MdR, SMW).

Sessions with individuals with aphasia lasted approximately 1.5–2 hours and were mostly completed at VUMC but in a few cases were conducted at the participant’s home (*n* = 4). All sessions with neurotypical controls were completed at the lab and took about 1 hour.

### Neuroimaging

Structural MRI or CT scans from each individual with aphasia were obtained for lesion delineation for lesion-symptom mapping (LSM) analyses. We generally drew the lesion on the scan closest in date to when individuals completed their behavioral testing, with a preference for MRI over CT scans, if available. For many individuals, lesions were drawn on chronic MRI or CT scans that were obtained for other research purposes in the lab or from follow-up neurology clinical care. For others, particularly those who were recruited from outside the Vanderbilt Stroke Center/Language Neuroscience Lab, only acute or subacute scans were available, so lesions were drawn on these. The time between scan and behavioral testing dates was 27.48 ± 17.38 months (range: 0–52 months; *n* = 25 MR, *n* = 8 CT images).

Lesions were manually delineated in ITK-SNAP on Linux workstations following an established protocol (Wilson et al., 2023). When MRIs were available, ischemic strokes were delineated in native space on diffusion weighted imaging (DWI) and hemorrhagic strokes on fluid attenuated inversion recovery (FLAIR) images; in both cases other imaging modalities were considered as needed for accurate lesion delineation.

Following lesion drawing, images were co-registered to the lesion mask and MRI images were segmented with reference to FLAIR images and warped to Montreal Neurological Institute (MNI) space using Unified Segmentation (Ashburner & Friston, 2005) and enantiomorphic normalization, where the lesion is replaced with intact tissue from the right hemisphere (Nachev et al., 2008). CT images followed the same approach. All lesion images were then smoothed with an 8 mm full-width half-maximum Gaussian kernel, thereby turning lesion status into a continuous variable.

### Statistical Analyses

#### Rhythm production data

Data were analyzed using circular statistics (Fisher, 1993). Commonly, tapping data in adults is analyzed using linear statistics which assumes relatively consistent tapping over the course of a trial and a small asynchrony (i.e., deviation) between a tap and the nearest stimulus. However, our aphasia cohort often had irregular and out-of-phase tap responses over the course of a trial. We thus applied circular statistics such that every tap response was included in analysis rather than only those that were within a small time window of the stimulus; our approach and rationale were informed by analyses of tapping data in children (Kirschner & Tomasello, 2009; Woodruff Carr et al., 2014). More broadly, circular statistics are ideally suited for capturing individual differences, a primary goal of this research. But, prior work in healthy adults has shown that circular and linear statistical approaches to analyzing tapping data give very similar results (Anglada-Tort et al., 2022; Dalla Bella et al., 2017; Fiveash et al., 2022; Niarchou et al., 2022).

Prior to formal analysis, all tapping (isochronous and music) trials for each participant were manually inspected for accurate tapping onset detection. In the majority of cases, onsets were automatically and correctly identified with pre-set parameters from the experimental MATLAB scripts (Anglada-Tort et al., 2022). However in some cases, onsets were not detected automatically despite them being easily identified as taps in the audio output recording. In these cases, manual modifications were made following a detailed pipeline and custom MATLAB scripts. Fisher’s exact tests revealed that the number of patients and controls that required any number of manual modifications were not different from one another in the isochronous task (odds ratio [OR] = 2.81, *p* = 0.075) nor in the music task (OR = 1.01, *p* = 1.00).

We analyzed our tapping data similarly to other tapping papers (Anglada-Tort et al., 2022; Dalla Bella et al., 2017; Fiveash et al., 2022; Sowiński & Dalla Bella, 2013) and use terminology for vector length and angle from the seminal work of Fisher (1993). For each tapping trial, we calculated a mean resultant vector *R*, which was computed from the distribution of all tap responses in polar coordinate space. The length, or radius, of *R* ranges from 0–1 and indexes synchronization consistency (1 = perfect synchronization, 0 = random tapping). The mean direction, or angle *θ*, of vector *R* indexes synchronization accuracy and ranges from −180° to 180° on a unit circle, where the stimulus occurs at 0°. Positive values indicate taps lagged behind the beat, while negative values indicate taps anticipated (i.e., were ahead of) the beat. While 0° is commonly used as the reference point for circular statistics tapping analyses (Fiveash et al., 2022; Kirschner & Tomasello, 2009; Sowiński & Dalla Bella, 2013), it is important to acknowledge that humans commonly anticipate the beat (i.e., producing negative mean angles) when synchronizing to an external cue (Repp, 2005). We also ran Rayleigh’s test for circular uniformity to assess whether tapping performance was above chance (Fisher, 1993; Wilkie, 1983), but did not exclude participants on the basis of this test, again similar to approaches in individuals with diverse tapping performance (Kirschner & Tomasello, 2009).

In the isochronous task, the first two taps for each participant were discarded from analysis; in the music task, taps were analyzed only after the metronome disappeared (Niarchou et al., 2022).

The first and second repetitions for each unique music clip were highly correlated with one another, both for vector length and angle. Thus, all subsequent analyses averaged the two repetitions for each unique musical clip, except in the minority of cases (*n* = 2 trials across all 62 participants) where only one repetition contained usable data, due to environmental interruptions during experiment testing.

Two principal component analyses, conducted separately for the aphasia and control groups, revealed that rhythm production was best explained by one component for each group (more details in Supplementary Data, Figures, and Tables). A tapping composite score for each participant was then computed, which was the average of vector length and vector angle across nine stimuli (3 isochronous, 6 music). Prior to averaging length and angle, vector length was stretched to be on the same scale (0–180) as vector angle, and vector angle was absolute valued and multiplied by –1 to reflect the same directionality as vector length (where more positive numbers indicate better performance) (Sowinski & Dalla Bella, 2013). A main reason for using a composite tapping measure is because we were most interested in creating a global measure of rhythm production, to be used in subsequent analyses with brain data, since tapping/beat detection has not been systematically assessed in an aphasia cohort of our size previously. Prior work in aphasia has also made use of a tapping composite score for dimensionality reduction to assess the relationship between rhythm production and other clinical measures (Zipse et al., 2014). Note that our composite measure treats individuals who were leading or lagging behind the beat as fundamentally similar, because of the absolute value transformation. The rationale for this approach was that we aimed to quantify whether each individual was struggling with tapping, rather than the exact manner in which they struggled, as the more clinically relevant outcome measure.

To identify impaired tappers, we performed Crawford-Howell’s *t* tests, which estimate the atypicality of a participant’s performance relative to a normative sample (Crawford & Garthwaite, 2012; Crawford & Howell, 1998). Control data were used as the normative data, excluding *n* = 1 control from this calculation based on visual inspection (i.e., control group mean was based on *n* = 28). Tests were one-tailed. We used a conservative *p* < 0.02 to identify impaired tappers (Casilio et al., 2024; Crawford et al., 2006). This value was empirically derived from simulation studies with small sample sizes (Crawford et al., 2006).

#### Rhythm perception data

BAT data were analyzed using signal detection theory with *d*-prime (*d*′) as the primary outcome measure (Stanislaw & Todorov, 1999). *d*′ is calculated by taking the *z*-transform (*norminv* in MATLAB) of the hit rate and subtracting from it the *z*-transform of the false alarm rate. In this case a hit was when an “on the beat’ stimulus was correctly identified as such and a false alarm was when an “off the beat” stimulus was mistakenly identified as on the beat. Extreme hit and false alarm rates of 1 and 0 were corrected to 0.99 and 0.01, respectively (Fiveash et al., 2020; Stanislaw & Todorov, 1999). We also computed percent accuracy and response bias *c* for each participant.

Similar to above, we categorized individuals as impaired/unimpaired on the BAT based on Crawford-Howell *t* tests (again excluding the same one control participant from this calculation).

#### Behavioral analyses

We fit multiple linear regression models with the *fitlm* function in MATLAB to assess (a) group performance in tapping and (b) the effect of various behavioral factors (motor, stroke, music training) on rhythm performance within the aphasia group. Effect size was measured using Cohen’s *f*^2^ (Cohen, 1988). Correlations were computed using Pearson’s *r*.

#### Lesion-symptom mapping analysis

Voxel-based LSM (VLSM) analyses were conducted with the *vlsm2* function in MATLAB (Bates et al., 2003; Wilson et al., 2010). At each voxel, a general linear model was fit to quantify the relationship between lesion status and behavioral outcome; analyses were conducted in voxels that were lesioned in at least five patients. Total lesion volume was always included as a covariate. Cluster-based permutation testing with 10,000 iterations was used to correct for multiple comparisons and only clusters with corrected *p* < 0.05 (right-tailed) were considered significant.

## RESULTS

### Comparison Between People With Aphasia and a Normative Sample on Measures of Rhythm Abilities

Descriptive statistics for all assessments are presented in Table 2. The two groups did not significantly differ on the MES Total score (*t*(60) = –0.81, *p* = 0.42, 95% CI = [–4.8, 2.03]), indicating that individuals with aphasia and controls were matched on amount of formal musical training.

**Table 2.** Descriptive statistics (mean ± std) for assessments

	Aphasia	Controls
Tapping	41.1 ± 28.4	63.7 ± 19.2
BAT *d*′	1.4 ± 1.3	3.0 ± 1.3
MES	13.0 ± 6.6	14.4 ± 6.8
QAB overall	6.4 ± 2.7	NA
NIHSS—right side	0.8 ± 1.1	NA
AST—left side	0.6 ± 0.3	NA

*Notes*. BAT = Beat Alignment Test; MES = musical experience survey; QAB = Quick Aphasia Battery; NIHSS = NIH Stroke Scale; AST = Apraxia Screen of TULIA.

**Figure 1.**
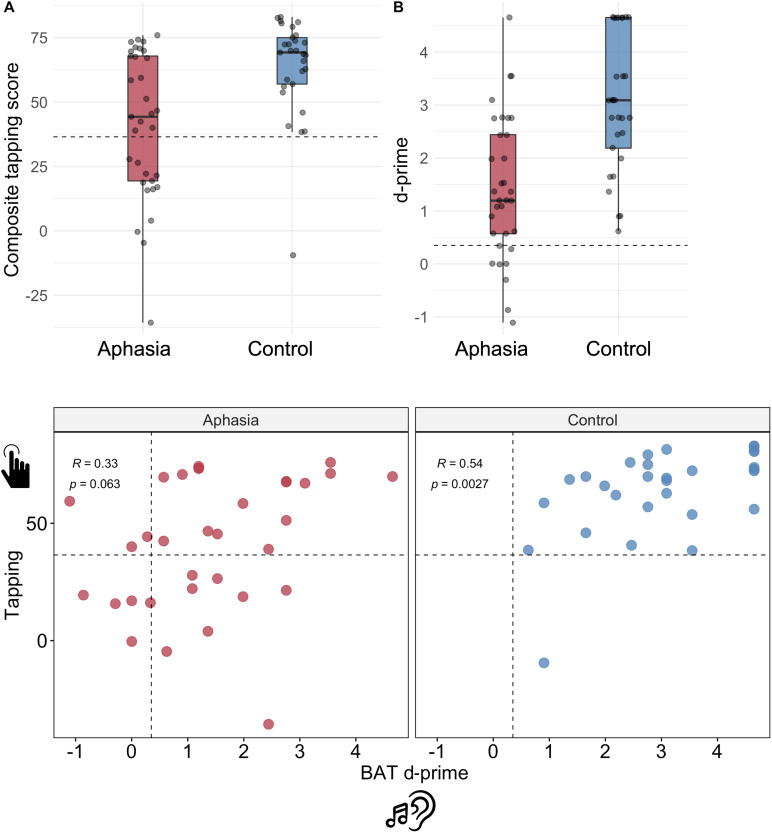
Tapping and beat perception group comparisons. Top: Group comparisons between (A) composite rhythm tapping and (B) beat perception. Each dot is a participant. Dashed lines indicate cutoff for rhythm production and perception based on Crawford-Howell *t* tests. Bottom: Correlations between composite tapping and BAT d-prime for each group. Dashed lines indicate cutoff scores. Aphasia group in red (left side), neurotypical control group in blue (right side). For visualization, points are jittered at jitter = 0.1.

Individuals with aphasia performed below the level of controls on tapping (*β* = 19.98, *p* = 0.0030, Δ*r*^2^ = 12.7%, *f*^2^ = 0.18) and demographic factors were not significantly associated with tapping (Table 3). The relationship between formal musical training and tapping performance was just statistically significant (*β* = 1.00, *p* = 0.047, Δ*r*^2^ = 5.46%, *f*^2^ = 0.075), although the two variables were not correlated in either group (Aphasia: *r* = 0.26, *p* = 0.14; Controls: *r* = 0.36, *p* = 0.057). In general, individuals with aphasia exhibited more variability in their tapping performance compared to controls (Figure 1 and Figure S1 and S2 for individual level data).

**Table 3.** Group differences in tapping

	*β* estimate	*SE*	*t*	*p* value	95% CI
Intercept	13.4	21.2	0.63	0.53	[−29.03, 55.91]
Group*	19.98	6.44	3.10	0.0030	[7.08, 32.87]
Age	0.13	0.20	0.68	0.50	[−0.26, 0.53]
Sex	3.56	6.67	0.53	0.60	[−9.81, 16.93]
Education	0.29	1.36	0.21	0.83	[−2.44, 3.02]
Handedness	8.45	8.36	1.01	0.32	[−8.31, 25.21]
MES*	1.00	0.49	2.04	0.047	[0.015, 1.98]

*Notes*. Model fit: *R*^2^ = 0.28; *F*(6, 55) = 3.48; *p* = 0.0054. Variable coding for binary variables: group (1 = controls, 0 = patients); sex (1 = female, 0 = male); handedness (1 = left-handed or ambidextrous, 0 = right-handed). *SE* = standard error.

* Denotes significant predictor.

Similarly to rhythm production, beat perception (i.e., BAT *d*′) differed between the two groups, with individuals with aphasia performing below controls (*β* = 1.6, *p* < 0.001, Δ*r*^2^ = 25.9%, *f*^2^ = 0.50). Formal musical training was a significant predictor of beat perception abilities (*β* = 0.11, *p* < 0.001) and contributed to a significant amount of model variance, with a large effect size (Δ*r*^2^ = 19.3%, *f*^2^ = 0.37). Demographic factors were not associated with beat perception. See Table S2 for full model results.

To assess convergent validity (i.e., the relationship among beat-based skills), we next correlated rhythm production and perception. In the full sample, rhythm production and perception were significantly correlated (*r* = 0.53, *p* < 0.001). The two modalities were moderately and marginally related in aphasia (*r* = 0.33, *p* = 0.063) but were significantly related in controls (*r* = 0.54, *p* = 0.0027). It is possible that with a larger cohort, rhythm production and perception would have tracked more closely; the current lack of correlation in the aphasia group was in part driven by one outlier participant (>7 * mean Cook’s distance) such that removing this individual indicated a relationship between the two modalities in the aphasia group (*r* = 0.45, *p* = 0.0092).

Per Crawford-Howell *t* tests, 13 individuals with aphasia and one control participant were impaired on rhythm production. Eight individuals with aphasia and none of the controls were impaired on rhythm perception. The next set of results aimed to parse individual differences within the aphasia group on tapping—the more clinically relevant rhythm ability. Parallel analyses with the BAT (VLSM, correlation with language measures) are presented in Supplementary Data, Figures, and Tables.

### Neural Correlates of Tapping

**Figure 2.**
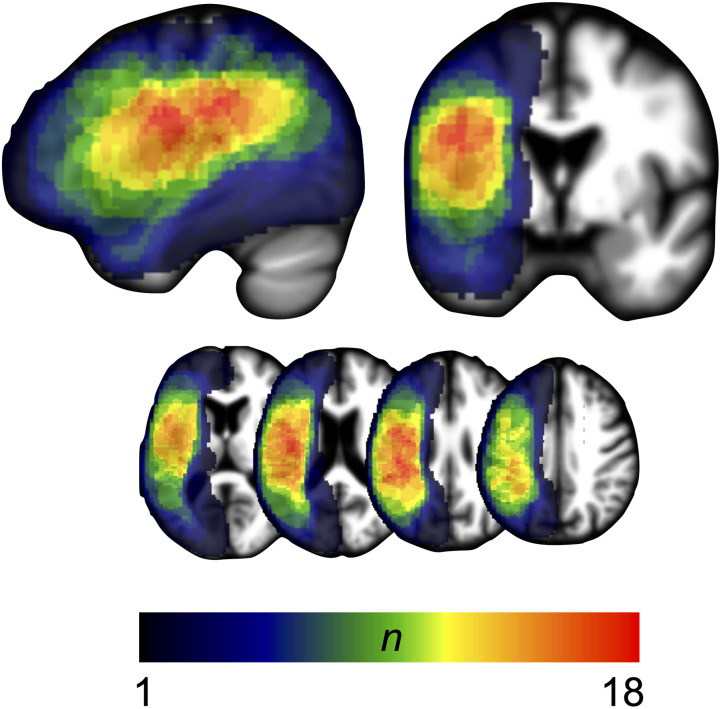
Lesion overlay. Lesion overlay for participants with aphasia (*n* = 33) in MNI space. Color bar shows number of patients with lesion in a given voxel, with hotter colors denoting greater degree of lesion overlap among patients.

A lesion overlay for all participants is presented in Figure 2. As the majority of participants with aphasia scored within the normal control range on tapping, we used the dichotomized scores for impaired/unimpaired tapping for subsequent LSM analyses; this approach has been used in other LSM analyses (Dressing et al., 2020; Martínez-Molina et al., 2022).

**Figure 3.**
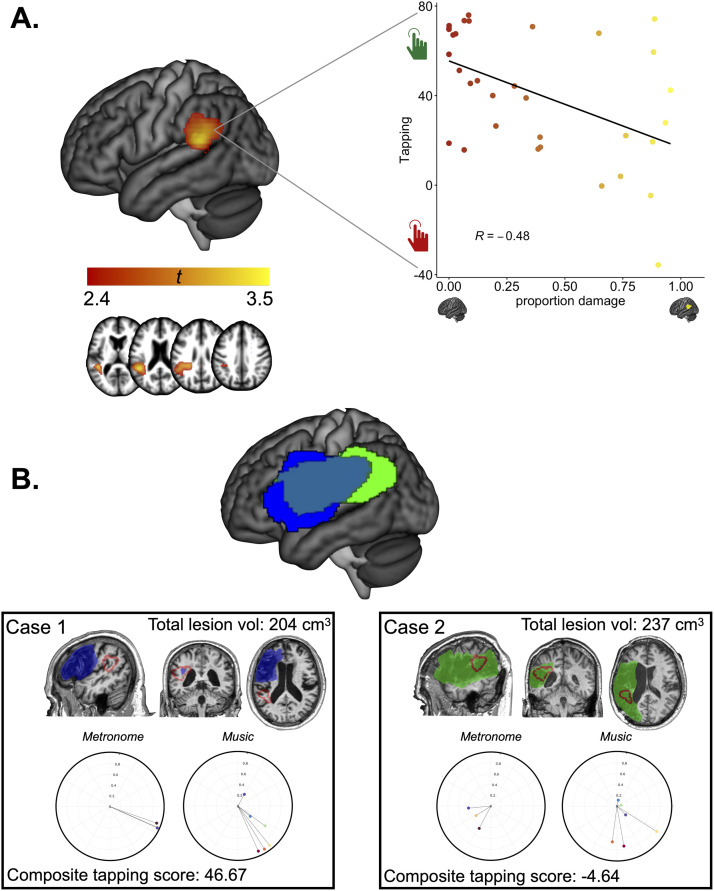
LSM results. (A) Results of LSM analysis, in MNI space. Values are voxelwise *t* values. Analyses were performed with a voxelwise threshold of *p* < 0.01 and corrected for multiple comparisons with a cluster threshold of *p* < 0.05. Side panel is correlation between proportion of damage to the significant LSM cluster and composite tapping scores. Each dot is a participant. Black line is the line of best fit. (B) Lesion overlay of impaired tappers (light green, *n* = 13) and unimpaired tappers (blue, *n* = 20) with overlap between the two groups in teal. A minimum of five patients in each group are plotted. Below are two representative participants with comparable lesion volumes; lesions are manually delineated according to group assignment (blue = unimpaired tapper, green = impaired tapper) and the ROI from our LSM outlined in red. Below are rose plots for individual tapping performance for all nine excerpts (3 isochronous, 6 music); each vector is an excerpt.

A lesion overlay of the impaired/unimpaired patient groups (Figure 3) revealed a partially overlapping distribution; however, the unimpaired group (*n* = 20) had a more frontal distribution while the impaired group (*n* = 13) had a posterior temporoparietal distribution.

The univariate LSM revealed one significant cluster (*p* = 0.036) in posterior superior temporal gyrus extending into supramarginal gyrus and the posterior segment of the arcuate fasciculus (AF) and superior longitudinal fasciculus (Table 4, Figure 3), suggesting that damage to this region is associated with poorer tapping abilities. As expected, we found that the proportion of damage to the region of interest from the whole-brain VLSM was correlated with the composite tapping score (*r* = −0.48, 95% CI = [−0.71, ‒0.17]), though damage to this region was neither necessary nor sufficient to result in rhythm impairments (Figure 3). That is, some individuals with considerable damage to this region performed quite well on tapping, while some individuals who had relative sparing of this region did not.

**Table 4.** Significant cluster for tapping VLSM

Region	Center of mass (MNI)	*t*	*p* value	Extent (mm^3^)
Posterior STG, extending into posterior segment of AF and into SMG	−43, −43, 24	3.46	0.036	19,384

*Notes*. White matter regions were identified with the aid of the XTRACT atlas (Warrington et al., 2020). STG = superior temporal gyrus; AF = arcuate fasciculus; SMG = supramarginal gyrus.

### Relationship Between Rhythm Production and Language

**Figure 4.**
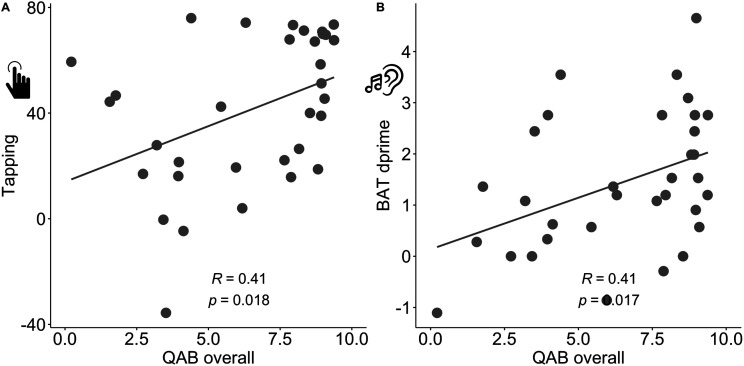
Rhythm and aphasia severity. Correlation between overall aphasia severity and (A) tapping composite and (B) beat perception.

Tapping correlated with overall aphasia severity (*r* = 0.41, *p* = 0.018), as did beat perception (*r* = 0.41, *p* = 0.017; Figure 4). Because of the high correlations among QAB summary measures and thus multicollinearity in these predictors (see Figure S3 and elsewhere in Supplementary Data, Figure, and Tables), we report exploratory correlations between tapping and each QAB summary score, without correcting for multiple comparisons and without including more than one language measure in a model. Language and motor scores for the impaired versus unimpaired tapping groups are presented in Table S3.

**Figure 5.**
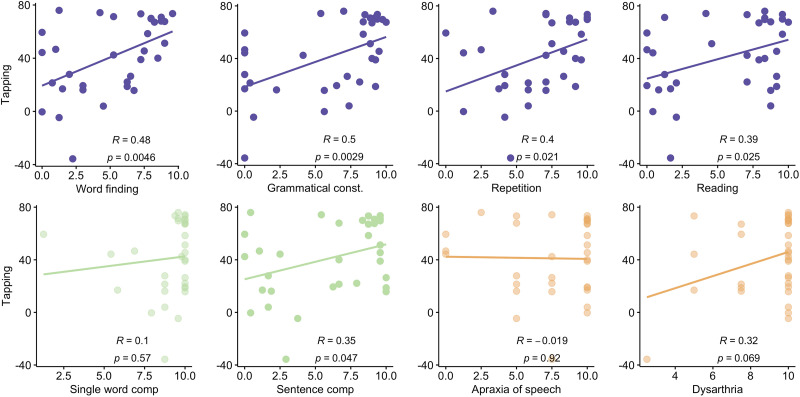
Tapping and language correlations. Scatter plots showing correlations between tapping and each QAB summary measure. Purple = language production; green = language perception; orange = motor speech. Panels with lighter shaded dots indicate correlations did not reach statistical significance.

Tapping correlated most strongly with several the QAB language production measures (word finding: *r* = 0.48, *p* = 0.0046; grammatical construction: *r* = 0.50, *p* = 0.0029). There were modest correlations with repetition (*r* = 0.40, *p* = 0.021), reading (*r* = 0.39, *p* = 0.025), and sentence comprehension (*r* = 0.35, *p* = 0.048), and no correlations between tapping and single word comprehension (*r* = 0.10, *p* = 0.57) or the motor speech measures (apraxia: *r* = −0.019, *p* = 0.92; dysarthria: *r* = 0.32, *p* = 0.069). Of note, the association between tapping and sentence comprehension was marginal. See Figure 5. In sum, this set of results—which must be considered exploratory—shows that some aspects of fluency, which is a multidimensional construct itself (Casilio et al., 2019; Clough & Gordon, 2020), were highly correlated with tapping (e.g., word finding and grammar), while speech motor programming, another component of fluency, was not. See Figure S4 for parallel results with the BAT.

### Behavioral and Clinical Predictors of Tapping Performance

Based on the AST cutoff scores, 21 individuals had limb apraxia and 12 did not (Figure S5). That is, most people in our post-stroke aphasia cohort also had limb apraxia.

Limb apraxia (as assessed on the left side, the side participants tapped with) was modestly related to tapping performance (*β* = 39.50, *p* = 0.041, Δ*r*^2^ = 11.7%, *f*^2^ = 0.15), but overall motor strength was not related (*β* = −6.03, *p* = 0.18; Table S4). Of the 13 impaired tappers, 12 of them also had limb apraxia. Beat perception was not significantly influenced by either motor variable (Table S4). This suggests that individuals with more severe motor planning and execution deficits were more impaired at tapping, and this was not a consequence of overall physical weakness.

Neither tapping nor beat perception were significantly associated with stroke covariates, including type of stroke (i.e., ischemic or hemorrhagic) or time post-stroke (Table S5).

## DISCUSSION

Taking an individual differences approach, we characterized rhythm abilities in depth in a sizeable cohort of participants with chronic, post-stroke aphasia. Focusing on tapping—the more clinically applicable rhythm skill—we found that there was marked individual variability in the aphasia group; some of this was explained by lesion location. Individuals who struggled with tapping tended to have damage to a left temporoparietal area extending into surrounding white matter. Individual variability in tapping abilities was also associated with language profiles; people who were less impaired overall, and on language production measures like grammar, tended to excel at tapping. A particular strength of this work is that one-third of participants with aphasia who participated had severe aphasia; many studies in chronic cohorts tend to recruit individuals who are moderate-mild. Our findings have prospective clinical applications for understanding which patients may respond best to rhythm-based interventions. These findings apply to the whole continuum of aphasia severity and provide mechanistic grounding for possible future treatment options for more severe individuals.

### Individuals With Aphasia Exhibit Variability in Rhythm

Most participants with aphasia were not impaired on rhythm; however, there was marked individual variability in patients and some variability in neurotypical controls. In the general population, people are known to vary in their rhythm abilities (Anglada-Tort et al., 2022; Niarchou et al., 2022) and auditory-motor synchronization skills at large (Assaneo et al., 2019; Mares et al., 2023). At a group level, individuals with aphasia performed below the level of controls on both rhythm tasks (similar to Zipse et al., 2014), which is unsurprising given they have experienced brain injury. In both this work and Zipse et al. (2014), about one-third of individuals with aphasia were classified as *rhythmically impaired*. However, it is important to note that the tasks in the two studies were not identical; Zipse and colleagues had participants tap to short, non-isochronous rhythmic sequences, rather than a metronome or excerpts of music.

Rhythm production and perception were associated with one another, albeit weakly in the aphasia group. Some prior studies suggest these may be separable skills (Fiveash et al., 2022) and that people can be impaired in one modality but not the other (i.e., work in *beat-deafness*; Bégel et al., 2017). Other work finds that production and perception are correlated and that (in modest sample sizes) paced tapping and the BAT perceptual task specifically are highly related (Dalla Bella et al., 2017; Iversen & Patel, 2008; Tranchant et al., 2021), which was replicated in the present study’s control cohort.

### A Dorsal, Posterior Temporoparietal Region Is Associated With Tapping

Impaired tapping was associated with damage to a left dorsal stream, perisylvian region at the crux of the temporal and parietal lobes, extending into the posterior segment of the AF, superior longitudinal fasciculus, and supramarginal gyrus. The significant cluster falls in a region long-known to be important for auditory-motor transformations (Kleist, 1962; Rauschecker & Scott, 2009); this region has also been termed *area Spt* by Hickok and colleagues. This area is a key part of the dorsal stream pathway for transforming sensory into motor representations (Hickok et al., 2003, 2009, 2011; Hickok & Poeppel, 2007). Along the dorsal auditory pathway, the inferior parietal lobule in particular is considered a pivot point for both forward and inverse mapping of sensory information and motor plans (Rauschecker, 2012; Rauschecker & Scott, 2009). This posterior perisylvian area is involved in sensory-motor processes including phonological encoding (Buchsbaum et al., 2011; Dickens et al., 2019; Henseler et al., 2014), but importantly is not speech specific (Hickok et al., 2003) and in functional imaging studies shows preference for sensorimotor processes involving the vocal tract (Hickok et al., 2011; Pa & Hickok, 2008).

Interfacing well with this work are hypotheses about the importance of the dorsal stream for beat perception and synchronization (Merchant & Honing, 2014; Patel, 2021; Patel & Iversen, 2014). These evolutionary-informed hypotheses posit that vocal learning served as a preadaptation for beat processing and that the neural circuitry for both overlaps along the dorsal auditory stream (Patel, 2021; e.g., posterior superior temporal regions, the posterior segment of the AF). The action simulation for auditory prediction (ASAP) hypothesis places particular emphasis on inferior parietal regions and underlying white matter (e.g., superior longitudinal fasciculus) for relating temporal predictions about beat timing (Patel & Iversen, 2014), and empirical work shows that coupling between dorsal auditory and motor regions underlies beat synchronization (Chen et al., 2006, 2008). Our work provides convergent evidence for the role of a left dorsal posterior region in nonlinguistic auditory-motor mappings, extending beyond the vocal tract and phonological encoding to beat synchronization.

Critically, the significant cluster from the VLSM falls just outside the bounds of canonical comprehension regions in the posterior temporal lobe (Henseler et al., 2014; Wilson et al., 2023), suggesting that our finding is not an artifact of comprehension. That is, people with damage to this area did not struggle with tapping due to possible lack of understanding of task directions. This is also corroborated by the fact that word and sentence comprehension did not strongly correlate with tapping.

We did not find that rhythm abilities were associated with damage to the basal ganglia (BG), as has been suggested in prior work (Stahl et al., 2011). Our results track more closely with studies of individuals with focal, unilateral BG lesions who do well at synchronizing with a beat (Aparicio et al., 2005; Schwartze et al., 2011). Patients with Parkinson’s disease exhibit some rhythm impairments (Cameron et al., 2016; Grahn & Brett, 2009), though in these studies participants are usually at a disease stage where BG damage is likely bilateral. A plausible hypothesis is that bilateral damage to BG structures manifests in rhythm impairments, but unilateral BG damage is not sufficient to cause notable rhythm deficits.

### Rhythm Is Associated With Language Production Measures But Not Motor Speech

Both rhythm perception and production were positively correlated with most of the language production measures (reading, repetition, grammar, word finding), in addition to overall aphasia severity. These results complement previous work in aphasia showing a relationship between speech output fluency and beat perception, extending now to the production modality. Our work also adds to the sizeable individual differences literature on musicality–language links in both children and adults (Nayak et al., 2022). In particular, there are several reported behavioral associations between beat-based processing and phonological skills (David et al., 2007; Grube et al., 2013; Moritz et al., 2013) as well as morphosyntax (Gordon, Shivers, et al., 2015; Nitin et al., 2023; Swaminathan & Schellenberg, 2020).

Dorsal tracts including portions of the AF are known to be important for syntactic processing (e.g., Wilson et al., 2011) which could be a plausible neural correlate related to the finding that tapping and QAB grammatical construction were especially related. This rhythm/syntax relationship may be mediated by prosody, as prosodic cues (e.g., duration and pitch information) facilitate understanding of hierarchical relationships in syntax (Fernald & McRoberts, 1996; Gordon, Jacobs, et al., 2015). The relationship between rhythm, prosody, and syntax warrants further attention, especially in older adults and those with focal brain injury, since the majority of work on this topic is in young children who are still acquiring language (Gordon, Shivers, et al., 2015; Ladányi et al., 2020; Nitin et al., 2023).

Tapping and apraxia of speech (AOS) were not associated. The lack of a relationship perhaps indicates that tapping is a relative strength in AOS (i.e., people with AOS are unimpaired at tapping such that this could be leveraged in treatment). This interpretation lends support to the use of techniques such as metrical pacing therapy and hypotheses about music-based interventions working best for individuals with motor speech disorders (Brendel & Ziegler, 2008; Zumbansen & Tremblay, 2019). Alternatively, an association might have been apparent in a cohort of participants with more variability in AOS profiles; in our sample, the distribution of AOS scores was positively skewed—half of the participants did not have any AOS. Though typical for a chronic aphasia cohort, this distribution may have masked a rhythm/apraxia association.

### Music Training and Limb Apraxia Differentially Influence Rhythm Perception and Production

Formal musical training was strongly associated with beat perception and less so with tapping. The latter is a fundamental human capability (Merchant et al., 2015) and is something humans can do quite well without any explicit training. The BAT on the other hand required participants to understand the concept of musical beat, and it is likely that individuals with previous musical training had a better grasp of this concept. Overall, there is mixed evidence on the relationship between tapping, beat perception abilities, and formal musicianship (Repp, 2010; Repp & Doggett, 2007; Tierney et al., 2021).

Limb apraxia had a modest influence on tapping performance when using continuous scores. However, all but one of the 13 impaired tappers also had limb apraxia, indicating there might be a tight association between the two when only looking at patients who really struggle with rhythm production; this observation is compatible with neural data. Limb apraxia is associated with lesions to the left inferior parietal lobule and posterior temporal and frontoparietal areas (Buxbaum et al., 2014; Buxbaum & Randerath, 2018), which partially overlaps with the present VLSM finding. While prior work has not found an association between rhythm production and limb apraxia in aphasia (Zipse et al., 2014), larger sample sizes are needed to further explore these relationships both behaviorally and neurally.

### Limitations

This work has several limitations. First and foremost, we cannot conclude anything about the role of the right hemisphere in rhythm processing directly from this study; only individuals with left hemisphere damage were recruited. While our results point toward a left posterior temporoparietal area, extending into white matter, that distinguishes impaired from unimpaired tappers, many studies using both lesion-deficit and fMRI methods suggest that the right hemisphere is involved in rhythm in some capacity, and also in music processing more generally (Albouy et al., 2020; Gordon et al., 2018; Kasdan et al., 2022). To fully answer questions about which brain regions are necessary and/or sufficient for rhythm, rhythm abilities would need to be assessed in patients with right hemisphere lesions and also patients with bilateral lesions, to understand any redundancy or degeneracy in the system (Price & Friston, 2002; Tononi et al., 1999).

Second, we used brain scans from various time points; for some individuals, only acute scans—acquired years prior to when behavioral testing occurred—were available, while for others, chronic scans were obtained within only a few weeks or months of testing. Lesions change over time (Gaudinski et al., 2008; Seghier et al., 2014). It is possible that for some individuals, their lesion may not have coincided with their behavior at the time of rhythm testing. Relatedly, LSM techniques generally only take into account areas of infarcted tissues. Other aspects of brain health, including leukoaraiosis and ventricle enlargement, are principally ignored in the lesion delineation process. Thus, certain neuroanatomical changes that can influence behavior, even those that are a consequence of stroke, are not captured in analyses. Together, these reasons, among others (e.g., diaschisis), are some of the challenges with current LSM methods as a whole (Seghier & Price, 2023).

Third, we used a composite tapping measure to analyze individual differences in rhythm production at a global, clinically meaningful level. Future work should explore tapping behavior at a more granular level, for example, isochronous clips versus music clips, deviations in vector length versus angle, tapping that is leading versus lagging, and so on.

Last, we used a mass univariate approach—VLSM—to answer questions about localization of rhythm processing. The drawbacks of such approaches have been discussed at length (Mah et al., 2014; Nachev, 2015). Future work could use multivariate LSM techniques such as support vector regression (DeMarco & Turkeltaub, 2018; Ivanova et al., 2021; Levy et al., 2024; Zhang et al., 2014) to make predictions about a patient’s rhythm abilities, which could complement localization findings.

### Clinical Implications

The finding that individual differences in tapping abilities may be due to the proportion of damage to a left posterior temporoparietal region fits well with some of the behavioral tenets of MIT but at the same time challenges ideas about it working through a right hemisphere mechanism. Our data suggest that the reason individuals with left posterior temporal lesions do not respond well to MIT is because they have damage to a core region important for rhythm production and auditory-motor transformations more generally. This explanation would also account for why individuals with global aphasia, who tend to have damage to the entire left middle cerebral artery territory, also do not respond well to MIT and why individuals with Broca’s/nonfluent aphasia, who tend to have damage predominantly to frontal regions, do respond well.

Converging with MIT is the work in speech entrainment for nonfluent aphasia; greater cortical activation has been found for speech entrainment compared to spontaneous speech in posterior parietal areas, and speech entrainment treatment-related changes were found in this same region (Fridriksson et al., 2012). These results support the current finding that a left posterior temporoparietal region is integral for nonlinguistic auditory-motor processing. It is possible that MIT, speech entrainment, script training, and related therapies with substantial rhythmic elements are operating through a spared left hemisphere mechanism, rather than through the right hemisphere. This hypothesis would align with the aphasia neuroplasticity literature at large which has found little convincing evidence for reorganization of language through the right hemisphere (Wilson & Schneck, 2021).

While it may be that the best candidates for rhythm-based interventions are those who do not have damage to our VLSM region, damage to this area was neither necessary nor sufficient to determine tapping abilities. It will be important for clinicians to assess rhythm skills directly (Curtis et al., 2019), in addition to utilizing brain imaging when available, to tailor aphasia therapies.

### Conclusion

The data presented here provides mechanistic groundwork for tailoring aphasia therapies, in line with precision medicine approaches. Speech-language pathologists should consider lesion location when making decisions about which patients would benefit from formal or informal rhythm-based therapies. Our work adds to both the historical literature on spared musical abilities in this population and anecdotal reports from speech-language pathologists about their use of rhythm in the clinic. Of course, any therapeutic approach should not only consider lesion information and abilities on experimental tasks, but should prioritize the personal goals of each individual without losing focus on improving functional communication and quality of life. Therapies such as script training have been shown to positively influence motivation and quality of life in people with aphasia (Cherney et al., 2008, 2022), and music therapies more generally have many emotional and psychosocial benefits (Särkämö, 2018; Tamplin et al., 2016). In addition to clinical applications, we hope this work also advances the understanding of the neurobiology of beat synchronization.

## ACKNOWLEDGMENTS

This work would not have been at all possible without the time and energy participants and their loved ones generously contributed. We also thank Dr. Sarah Schneck, Jillian Entrup, Caitlin Onuscheck, Dominique Herrington, Kelly Fussman, Zachary DeWall, and Jennifer Kile for support with patient recruitment; Catherine Bush and Lily Bronson for assisting with control participant recruitment; and Dr. Adeen Flinker, Dr. Miriam Lense, and Dr. Mark Wallace for input on analyses.

## FUNDING INFORMATION

Anna V. Kasdan, National Institute on Deafness and Other Communication Disorders (https://dx.doi.org/10.13039/100000055), Award ID: F31 DC020112. Anna V. Kasdan, National Science Foundation Graduate Research Fellowship Program (https://dx.doi.org/10.13039/100023581). Marianne Casilio, National Institute on Deafness and Other Communication Disorders (https://dx.doi.org/10.13039/100000055), Award ID: F31 DC021108. Deborah F. Levy, National Institute on Deafness and Other Communication Disorders (https://dx.doi.org/10.13039/100000055), Award ID: F32 DC020096. Michael de Riesthal, National Institute on Deafness and Other Communication Disorders (https://dx.doi.org/10.13039/100000055), Award ID: R01 DC013270. Reyna L. Gordon, National Institute on Deafness and Other Communication Disorders (https://dx.doi.org/10.13039/100000055), Award ID: R01 DC016977. Stephen M. Wilson, National Institute on Deafness and Other Communication Disorders (https://dx.doi.org/10.13039/100000055), Award ID: R01 DC013270.

## AUTHOR CONTRIBUTIONS

**Anna V. Kasdan**: Conceptualization; Data curation; Formal analysis; Funding acquisition; Investigation; Methodology; Project administration; Visualization; Writing – original draft; Writing – review & editing. **Marianne Casilio**: Conceptualization; Formal analysis; Investigation; Methodology; Writing – review & editing. **Katherine Bryan**: Data curation; Formal analysis; Investigation. **Nori Jacoby**: Conceptualization; Formal analysis; Methodology; Writing – review & editing. **Noah R. Fram**: Methodology; Validation; Writing – review & editing. **Lily Walljasper**: Formal analysis; Investigation; Project administration. **Deborah F. Levy**: Conceptualization; Validation. **Michael de Riesthal**: Methodology; Resources; Validation. **Reyna L. Gordon**: Conceptualization; Funding acquisition; Methodology; Resources; Supervision; Writing – review & editing. **Stephen M. Wilson**: Conceptualization; Formal analysis; Funding acquisition; Methodology; Resources; Supervision; Validation; Writing – review & editing.

## DATA AVAILABILITY

De-identified data and MATLAB code for the main analyses are available at the following Open Science Framework (OSF) repository: osf.io/23qub. Aphasia-friendly materials used in the research study are available in Supplementary Materials 2 and 3.

## Supplementary Material









## TECHNICAL TERMS

Aphasia: Acquired communication disorder resulting from damage to language regions of the brain.
Beat: Context in which musical rhythms are typically perceived; a “regular pulse” that can be organized hierarchically.
Musical training: Musical experience and practice, for example, through music lessons, orchestra, band, choir, or playing an instrument.
Lesion-symptom mapping: Technique used to investigate relationship between patterns of brain damage and behavior(s) of interest.
Circular statistics: Statistical method used in the current manuscript to analyze tapping data; allows for all tap responses to be included in analysis and is ideally suited for capturing individual differences.
