# Supplementary material for: Neural Correlates of Rhythm in Post-Stroke Aphasia
Source: Neurobiol Lang (Camb). 2025 Aug 14;6:nol.a.9. doi: 10.1162/nol.a.9 (PMC12373457; doi:10.1162/nol.a.9)

Tapping to a beat in aphasia

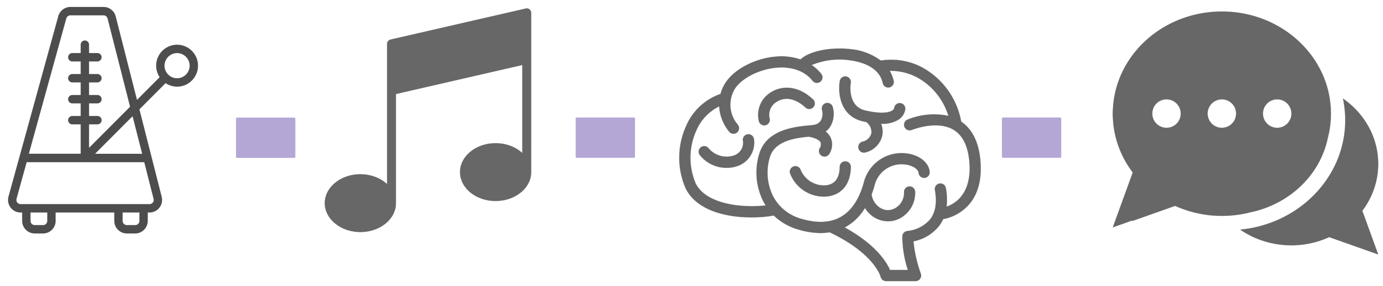

by: Anna V. Kasdan, Marianne Casilio, Kat Bryan, Nori Jacoby, Noah R. Fram, Lily Walljasper, Deborah F. Levy, Michael de Riesthal, Reyna L. Gordon, Stephen M. Wilson

An accessible version of "Neural correlates of rhythm in post-stroke aphasia" designed for individuals with aphasia and their loved ones

Abstract/Summary

- **Aphasia** is a **problem** with **language** that can happen after **stroke**

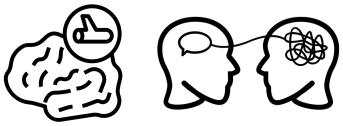

- **Tapping** to a **beat** can help people with aphasia get the **words out**, but we don’t know exactly who this **works best** for

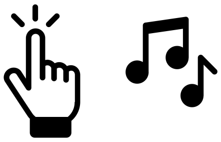

- A group of **people with aphasia** and a group of **people without aphasia** did our research study

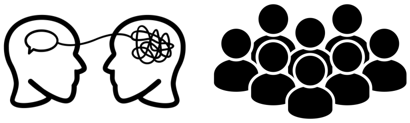

- We found that many people with **aphasia** were **good** at tapping!

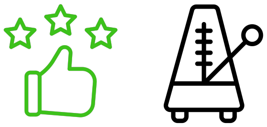

- People who **struggled** tended to have their **stroke** in a part of the brain called the **left temporal** lobe

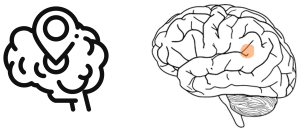

- We **hope** our research will **help speech therapists** decide **which patients** they should try rhythm and **tapping therapies** with

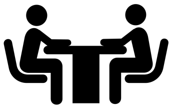

Introduction (1):
What we know already

- **Aphasia** is a **problem** with **language** that can happen after **stroke**

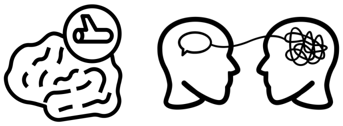

- People with aphasia are usually still **good at musical activities**, like singing or tapping to a beat, even when they have trouble talking

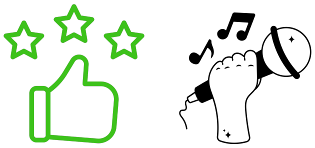

- **Speech therapists** often **tap** and use musical **rhythm** with people with aphasia to **help** them get the **words out**

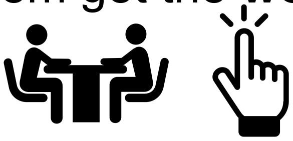

- This **works** for **some people**, but **not everyone**

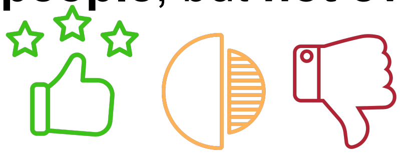

- Even though rhythm is used all the time in speech therapy, there is **not a lot of research** about it

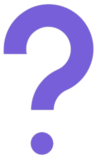

Introduction (2): What we wanted to know

- We know that the **location of** a **stroke** in the brain **affects** the types of **language** problems people have

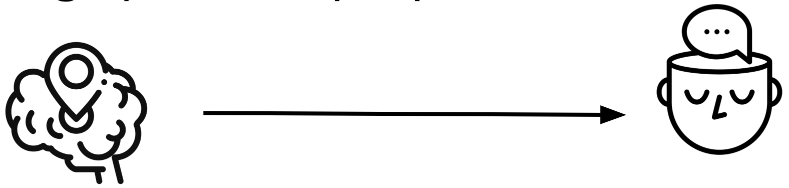

- So, does the **location** or size of a stroke also affect their **tapping**?

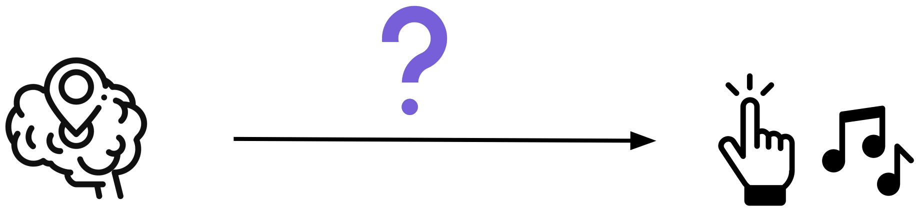

- How do people with more **severe aphasia and** specific **types** of **language problem**s do at **tapping**?

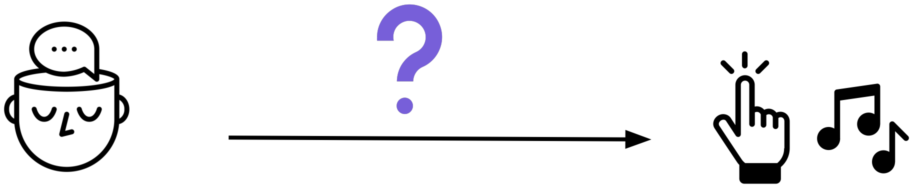

- Is it **easy or hard** for people with aphasia to keep a beat?

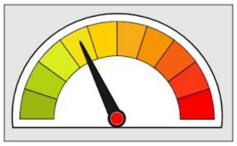

- Knowing this would **help speech therapists** decide **which patients** they should try **rhythm** and tapping **therapies** with

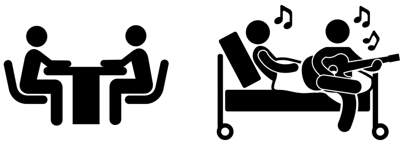

Introduction (3):
What we did in this research study

- We had many people with aphasia do **rhythm activities** in the lab

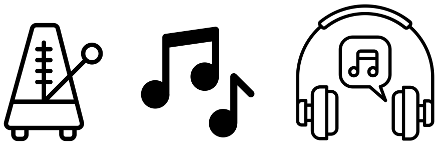

- We got information about everyone's **strokes**

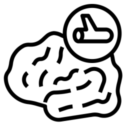

- This is something **not many scientists** have done **before**!

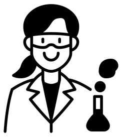

Methods (1):
Who did the research study?

- We saw **33 people** who had **aphasia** because of a **stroke**

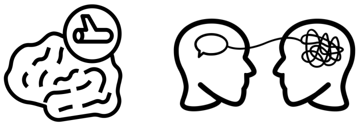

- Everyone with aphasia had their **stroke** at least **one year ago**

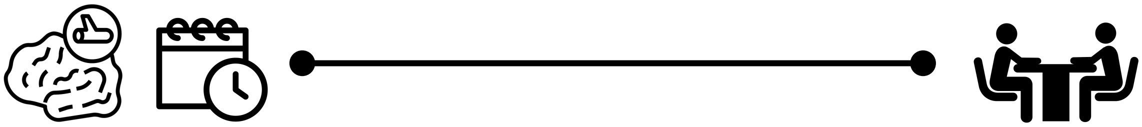

- We also saw **29 people** who did **not have aphasia**
    - This group was called the **control group** 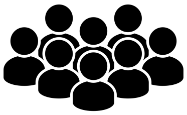

- Everyone **agreed** to be in the research study 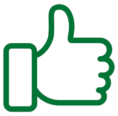

Methods (2):
What did people do?

- Everyone did **music tasks** which included:
  - Tapping to a **metronome**, which is a steady beat 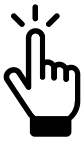 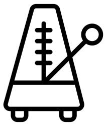
  - Tapping to the beat of **music** 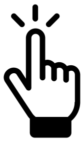 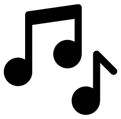
  - **Listening** to musical rhythms 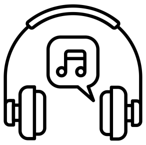
  - A short **survey** about **musical background** and experiences

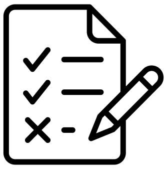

Methods (3):
What else did people with aphasia do?

- Everyone also did a **language test** called the Quick Aphasia Battery (QAB)

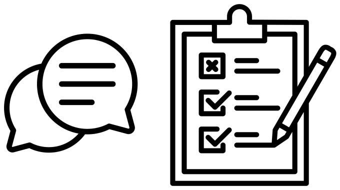

- Sometimes, people struggle with **movement after stroke**. So, we also tested:
    - **Arm strength** 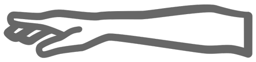
    - **Muscle coordination** (“limb apraxia”) 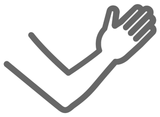

Methods (4):
How we got information about the brain

- We had **pictures** of people's **brain** so we could see their stroke

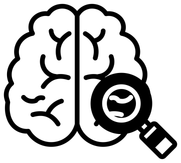

- We used a computer program to **highlight** their **stroke**

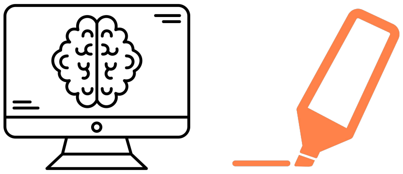

- Then we used **computer code** to look at whether the **location** of their **stroke** impacted how they did at **tapping**

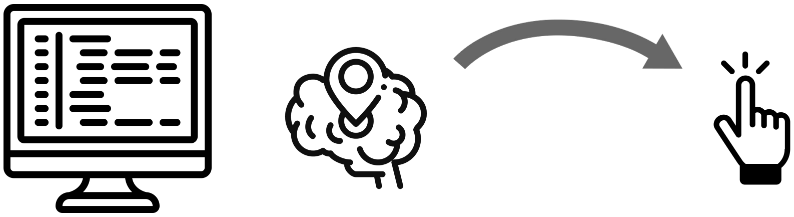

Results (1):
Comparing people with aphasia and controls

- Overall, **people without aphasia** had **higher tapping scores** than people with aphasia

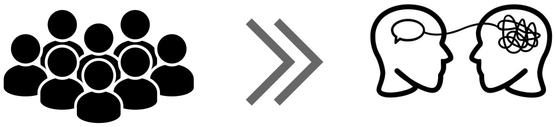

- However, many **people with aphasia** did **well** at tapping

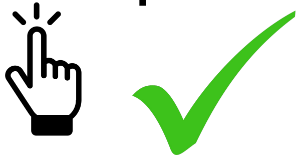

- But, **some** people with aphasia still **struggled** with tapping

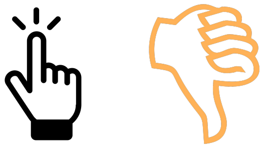

- So, **why** do some people with **aphasia** have **difficulty tapping**, but others do not?

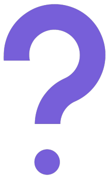

Results (2):
Analyzing the brain and tapping

- People with strokes in a specific part of the **left temporal lobe** had more difficulty keeping the beat

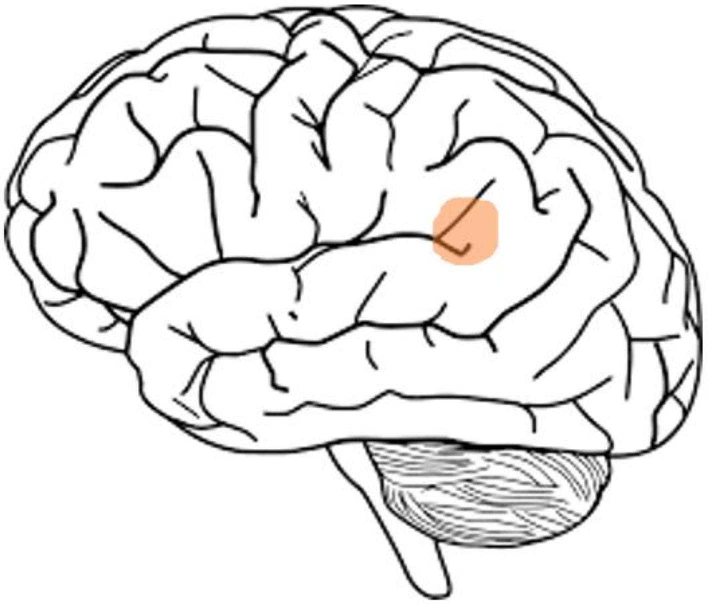

- This area included gray and white matter in the brain

- **Not everyone** who had a stroke in this part of the brain had trouble tapping, **but many** people did

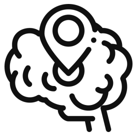

Results (3):
Other factors matter for tapping in aphasia

- **People with aphasia** who had an **easier time tapping** *usually* also:
  - Had **higher language and talking** abilities

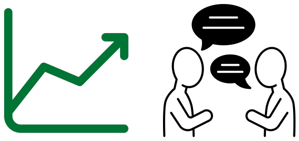

  - Were **good at coordinating movements** and did not have limb apraxia

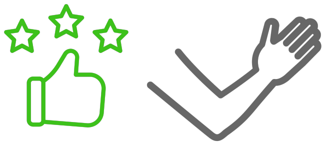

- **Music training didn't make** a big **difference** in how people did on tapping

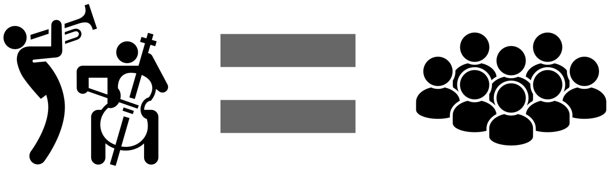

Discussion (1):
Main ideas

- This study helps us **understand** how people with aphasia do at **tapping**
    - **Many** people were quite **good** and **some performed** just **like people without aphasia**!
- This research is important because it helps us know **who may respond best to rhythm** and tapping exercises during speech therapy
    - People who have strokes in **left frontal** areas may really benefit from **tapping** therapies

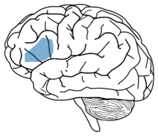

    - People who have strokes in **left temporal** areas may do better with **different** types of therapies

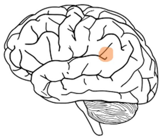

- Information about **stroke location** in the brain is important

Discussion (2):
Why this study is important

- **Before**, there was **not much research** on this topic even though speech therapists use tapping a lot

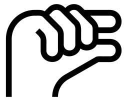

- **Now**, we **know more** about who exactly rhythm might be best for!

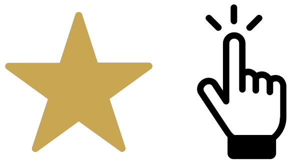

- This work can **help** people with **aphasia,** their **loved ones**, and **clinicians**

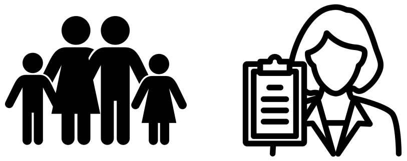

- **Many** people with **severe aphasia participated**!
  - Usually, studies don't include many people with severe language difficulties, since the research activities can be too hard for them

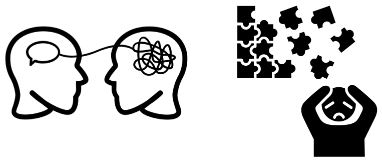

Discussion (3):
Limitations/Things to talk about

- We **only studied** people who had **strokes** in the **left** hemisphere of the brain
  - Other parts of the brain on the **right** side might be important for tapping

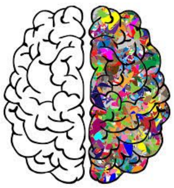

- How a **stroke** looks on a brain image **can change** over **time**
  - Next time, we could get **everyone's newest** brain image

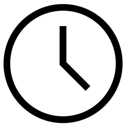 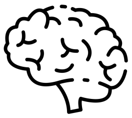

- There are **many ways** to **analyze** the brain and tapping data
  - Some **scientists** may use **different methods** and types of computer code, so may get slightly different results

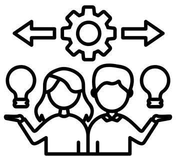 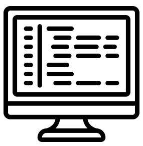

Conclusion

- For some people with aphasia, tapping to a beat could be a **helpful tool** for them to get the **words out**

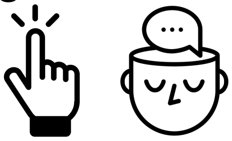

- The **location** of the **stroke** was important for understanding who might struggle with tapping

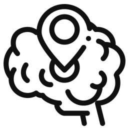

- There are also **other factors** besides the brain that **mattered**, including someone’s movement abilities!

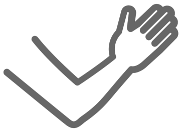

- We **hope** our work will **help speech therapists** understand who with aphasia might respond well to rhythm and tapping therapies

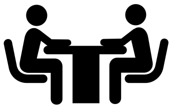

- Of course, there is still a lot **more research** to do

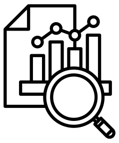

- **Thank you** to everyone who helped with our research - we couldn’t have done it without you!

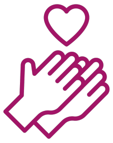

Supplement: Supplementary file 1 [file nol-6-1-9-s001.pdf]
