# Supplementary material for: Neural Correlates of Rhythm in Post-Stroke Aphasia
Source: Neurobiol Lang (Camb). 2025 Aug 14;6:nol.a.9. doi: 10.1162/nol.a.9 (PMC12373457; doi:10.1162/nol.a.9)
Supplement: Supplementary file 2 [file nol-6-1-9-s002.pdf]

Informed Consent

This informed consent document applies to adults with aphasia.

Key Information

Who is in charge of this study?

Anna Kasdan, student PI

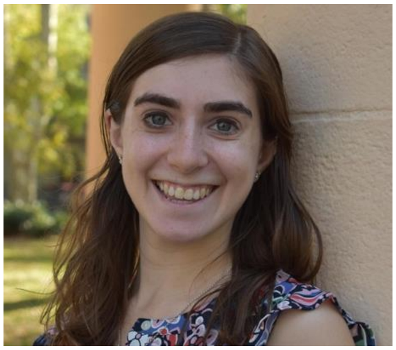

Dr. Stephen Wilson, faculty advisor

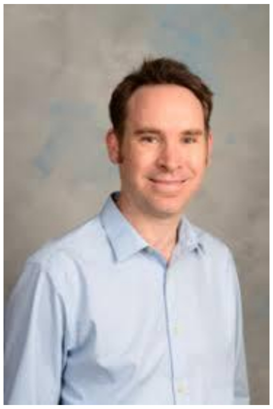

What is the study called?

The study is called "Brain basis of rhythm in neurological patients and neurotypical individuals".

Where is the study happening?

Vanderbilt University Medical Center

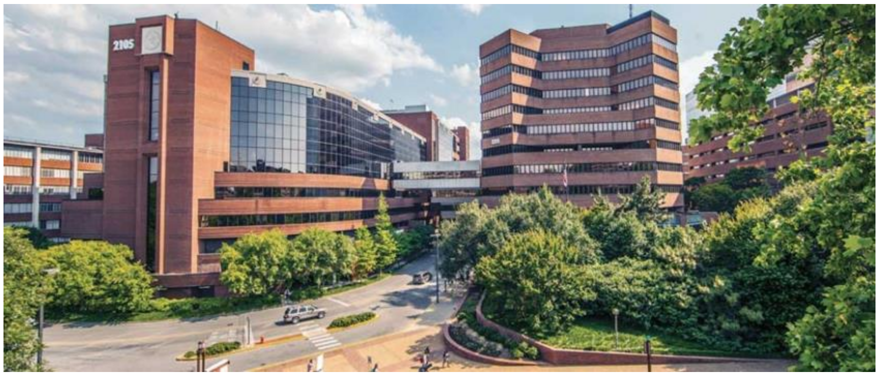

What is this booklet?

This booklet is meant to help you understand our research study.

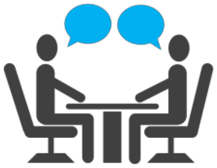
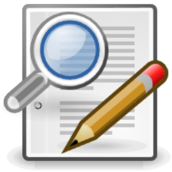

Please ask questions!

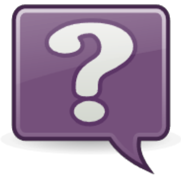
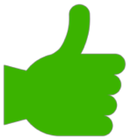

We will give you a copy of this booklet.

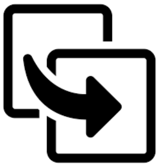

What is this study about?

We want to learn about your music and language abilities since your aphasia.

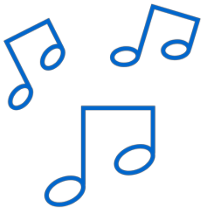
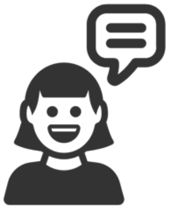
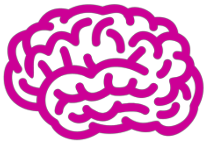

Detailed Information

We are asking you to be in our study because you have aphasia.

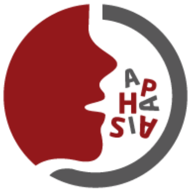

We will ask you to complete some language and music assessments. We may also ask you to complete some surveys and tasks on the computer.

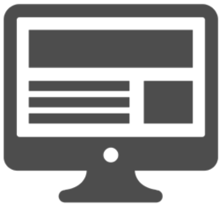

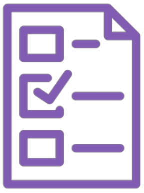

We will ask to film the study session.

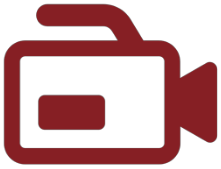

We will also ask for your medical recordings relating to your aphasia if that is OK with you. This is so that we can better understand how your brain process music and language.

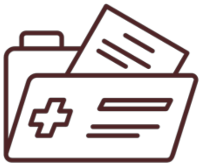

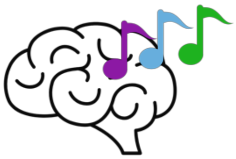

You do not have to be in the study.

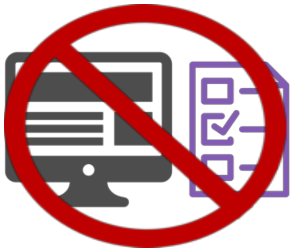

You can stop being in the study at any time.

If anything happens that might make you change your mind about being in the study, we will tell you. Then you can decide whether to stay in the study.

What will I have to do?

We will do a language assessment with you where you may be asked to talk, read, write, and listen to speech. You may also complete some short surveys.

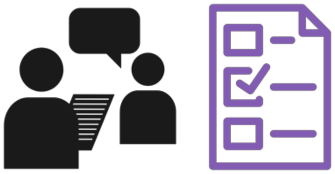

You will also do some music, and other related, tasks on the computer.

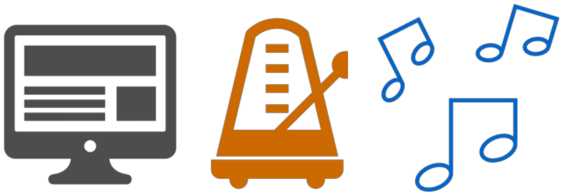

All of these tests will happen in one session. The session will be no longer than 2 hours and will probably take closer to 1 hour.

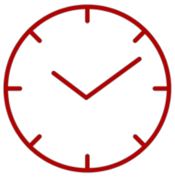

1 < 2 hrs.

You can stop or leave the study at any time.

Does it cost anything?

It does not cost anything for you to be in the study.

Are there any risks?

There are no unforseeable risks to this study.

What good might come of this study?

This study might help us understand how music and language processing is affected after aphasia.

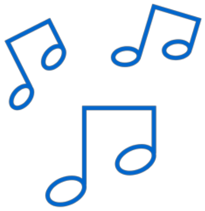
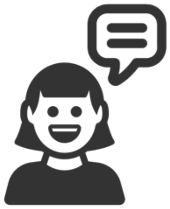
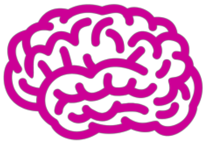
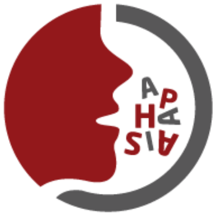

We don't know if this study will help you specifically, but the study might give you information about your music and language abilities.

Will I see the results of the study?

Yes! If you want to know how you did on the tasks in this study, we can meet with you again or send you a letter.

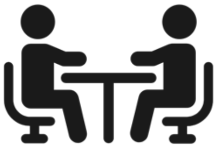
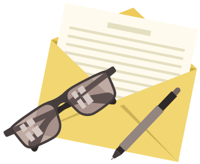

We will also present the results at scientific conferences and write about them for scientific journals.

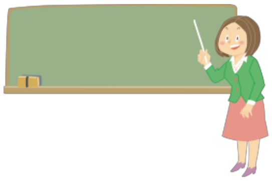
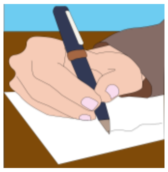

Is this a treatment study?

This is not a treatment study.

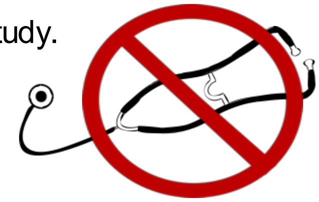

Will I get paid?

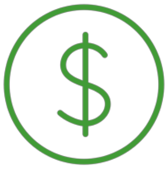

Yes! You will get paid $20/hour in gift card format.

If the study goes longer than 1 hour, you will be compensated accordingly (i.e., $30 for 1.5 hours, $40 for 2 hours, etc.).

Could I be taken out of the study?

We might take you out of the study if it seems like the study is making you upset, or if it gets harder for you to understand or think clearly.

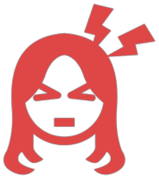

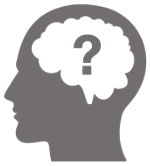

If we take you out of the study, we will let you know why.

What if I want to stop being in the study?

You can stop being in the study at any time.

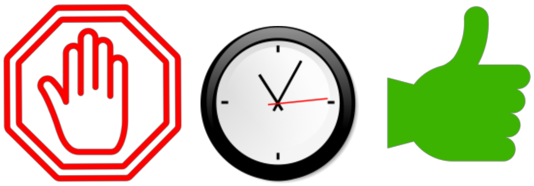

Who can I contact with questions?

If you have questions about this research study, you can…

Call: Anna Kasdan at [redacted]

Email: Anna Kasdan at anna.v.kasdan@vanderbilt.edu

OR, you can contact the faculty advisor, Dr. Stephen Wilson:

Call: Dr. Stephen Wilson at [redacted]

Email: Dr. Stephen Wilson at stephen.m.wilson@vumc.org

If you have concerns about the study, you can…

Call: The IRB office at (615) 322-2918
Call toll-free: The IRB Office at (866) 224-8273

Will anyone besides the researchers see my information?

We will do our best to keep your information private.

Your music and language assessments, videos, medical records, and other information will be stored on a password-protected server. Only people involved in the research study can see that information.

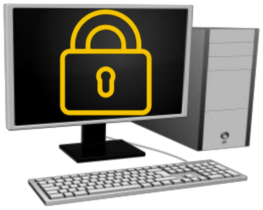

Some of the information we collect can't be linked back to you. We might share this information with other researchers. No one who sees this information will know it came from you.

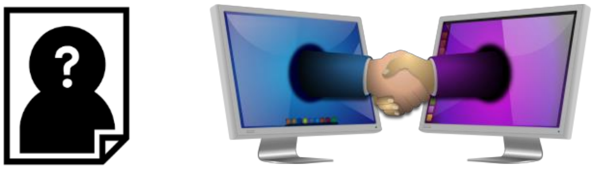

Videos of you can be matched with you because your face and voice are in them. You can choose how we use those videos.

Is it okay for us to…

______ Share videos of you in presentations or teaching materials?

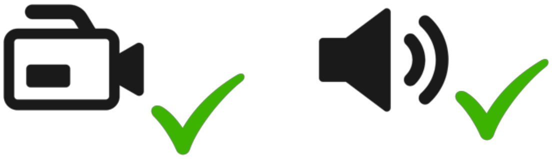

If not, choose one of these options:

______ Share only my audio for presentations or teaching materials.

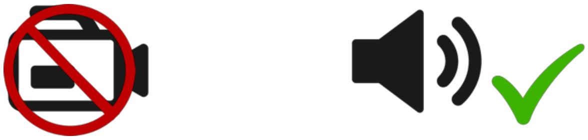

______ No one besides the researchers can see and hear the videos.

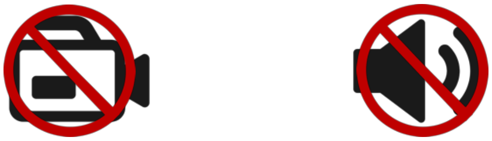

How will you get a hold of my medical records?

To do this research, we will need to access and use your medical records. These records contain your private health information.

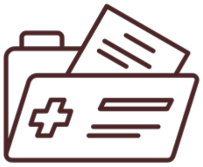
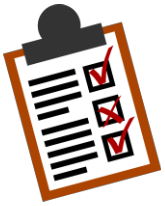
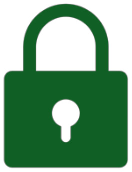

Your doctors and other health care providers will send us your medical records so that we can conduct the research study.

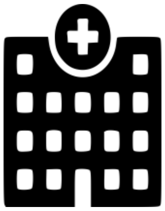
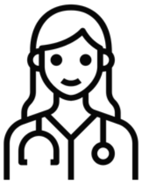
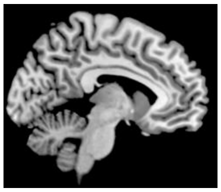

Only members of the research team will see this information. However, we may share this information with other providers, government agencies, or data managers. We try our best to keep everything confidential.

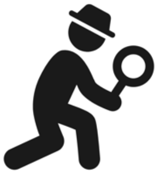
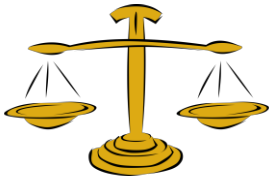
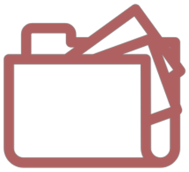

Once your medical records are shared with us, we will always have them. It does not expire.

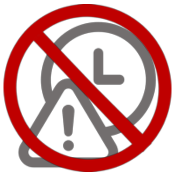

However, if you change your mind, you can tell us in writing to stop using your medical records. This will not affect information we collected for the research study before you changed your mind.

Your treatment, health plans, or involvement in any community groups stays the same and is not affected by this research study.

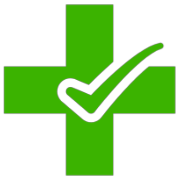
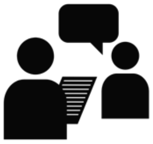
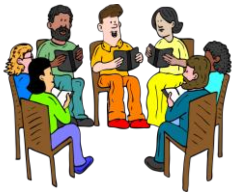

STATEMENT BY PERSON AGREEING TO PARTICIPATE IN THIS STUDY

I have **read** and **understood** the consent booklet.

I have had time to **think** and **ask questions.**

All of my **questions** were **answered.**

I am **choosing** to **be in** this **study.**

____
Date

____
Signature of volunteer

Consent obtained by:

____
Date

____
Signature

____
Printed Name and Title
