# Supplementary material for: Neural Correlates of Rhythm in Post-Stroke Aphasia
Source: Neurobiol Lang (Camb). 2025 Aug 14;6:nol.a.9. doi: 10.1162/nol.a.9 (PMC12373457; doi:10.1162/nol.a.9)
Supplement: Supplementary file 3 [file nol-6-1-9-s003.pdf]

Musical Experience Survey

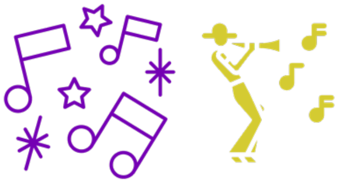

PID: ________________________
Date: ________________________
Experimenter(s): ________________________

1. Did you play an instrument as a **child**?

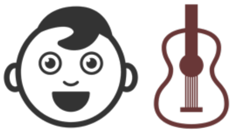

Yes ☐ ✔ No ☐ ✘

*If yes, how many **years** did you play?*

---

2. Do you **currently** play an instrument?

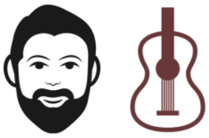

Yes ☐ ✔ No ☐ ✘

*If yes, how many **hours** a week do you play?*

---

3. Please indicate which **instrument(s)** you play/ever have played.

Piano/keyboard ☐

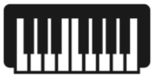

Recorder ☐

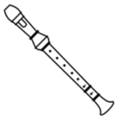

String instrument (violin, viola, cello) ☐

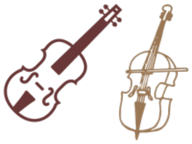

Percussion ☐

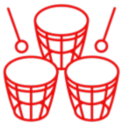

Guitar/bass ☐

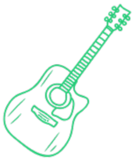

Woodwind instrument (flute, saxophone) ☐

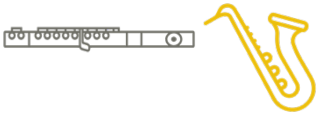

Brass instrument (trumpet, French horn, tuba, etc.) ☐

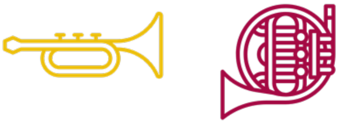

Voice/singing ☐

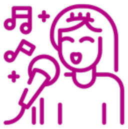

Institutional Review Board

VANDERBILT

4. Did you participate in any formal **musical training** (lessons, concert band, orchestra, choir, etc.)?

Yes ☐ No ☐

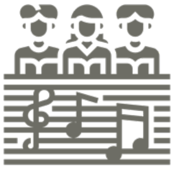

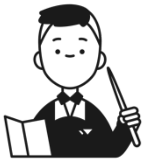

---

5. Did you participate in any formal **dance** training?

Yes ☐ No ☐

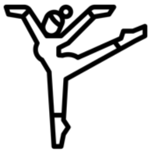

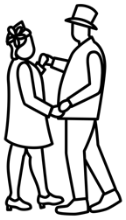

*If yes, for how many **years**?*
