# Supplementary material for: Neural Correlates of Rhythm in Post-Stroke Aphasia
Source: Neurobiol Lang (Camb). 2025 Aug 14;6:nol.a.9. doi: 10.1162/nol.a.9 (PMC12373457; doi:10.1162/nol.a.9)
Supplement: Supplementary file 4 [file nol-6-1-9-s004.pdf]

Supplementary Document 4 for Kasdan et al., "Neural bases of rhythm in post-stroke aphasia"

MES scoring procedures

Scoring decisions for our new musical experience survey (MES) for people with aphasia are described. Sometimes, participants provided a response on the MES that spanned two bins from the Goldsmiths Musical Sophistication Index (GMSI). In these cases, the higher score (i.e., more years of training, more hours of practice, etc.) was systematically given. In other instances, individuals were not specific about the number of years of musical training (MES Q1), in which case a score was assigned based on other qualitative information, such as details provided during the Quick Aphasia Battey (QAB) connected speech section or information provided by a loved one. Several individuals in both the aphasia and neurotypical control groups noted participating in weekly church choir – this was imputed to a score of 5 (2 hrs/week) for everyone.

Apraxia Screen of TULIA (AST) items and test administration

There were a few limitations with the items and test administration for this assessment. First, partway through data collection, we changed one imitation item – "use a stamp to postmark" – to "use a key to open a door." This "stamp" item was dated and ambiguous, especially given the face masks worn during testing, and participants found it confusing, so we changed the item for these reasons. N=12 participants received the "stamp" item. A Fisher's exact test indicated that there was no difference in performance between those who received the "stamp" vs "key" version of this item (OR=6.77, p=0.11, left side). Additionally, partway through data collection the research team expounded upon the test instructions for items 3-7 with an example cue item. The verbal instructions were not straightforward, especially for people with aphasia, and we found that adding an example cue for "how to use a fork" seemed to clarify the task. Half of the participants (n=16) did not receive this cue; however, their performance on items 3-7 was not significantly different from participants who did receive this additional cue (t(31)=0.62, p=0.52, 95% CI: [-0.64, 1.23], left side).

Principal component analysis (PCA) on rhythm production data

In each PCA (one for the aphasia group, one for the control group) there were 18 variables: vector length for all stimuli (3 isochronous, 6 music), and vector angle for all stimuli. To place the vector length and angle measures on the same scale, variables were z-scored prior to analyses, which were then performed using *pca* in MATLAB. Rhythm production was best explained by one component for each group (Aphasia: 41.7% variance explained, eigenvalue=7.5; Controls: 48.4% variance explained, eigenvalue=8.7). We also computed the variance inflation factor (VIF) to assess

multicollinearity for each group's tapping data. This revealed that the majority of predictors were highly correlated (VIF>5), further warranting a summary/composite score. The composite score calculation – described in the main text – was computed from the actual values for vector length and angle, and not from the *z*-scores that were used exclusively for conducting the PCA.

PCA on QAB summary measures

A PCA on the QAB summary measures (excluding the dysarthria component) from the 33 participants with aphasia showed that the QAB is best explained by one component (78.3% variance explained, eigenvalue=64.2). Most measures were highly correlated with one another (Figure S3) with VIF>5 in all cases.

Voxel-based lesion-symptom mapping (VLSM) with beat perception

VLSM analyses with the BAT did not reveal any clusters that survived correction for multiple comparisons, either using continuous (p=0.18) or cutoff (impaired/unimpaired) scores (p=0.37).

Beat perception, language, and motor abilities

Beat perception correlated with both language perception measures (single word comprehension: r=0.44, p=0.010; sentence comprehension: r=0.35, p=0.046), three of the four the language production measures (word finding: r=0.34, p=0.054; grammatical construction: r=0.39, p=0.026; repetition: r=0.47, p=0.0061; reading: r=0.37, p=0.032), and neither of the motor speech measures (apraxia: r=0.19, p=0.30; dysarthria: r=0.08, p=0.66), similarly to tapping. As the BAT required verbal explanation on the part of the experimenter and understanding of auditory directions on behalf of the participant, it is likely that the correlations with comprehension indicate that performance on the task was in part influenced by how well participants understood the task instructions.

Table S1 – Music excerpts used in tapping task

Track	Artist	Lyrics (Y/N)
You're the First, the Last, My Everything	Barry White	Y
Passe and Media, Den Iersten Gaillard	Susato – composer; Capilla Flamenca – group	N
Hurricane	Bob Dylan	Y
St. Matthews Passion, BWV 244	J.S. Bach	N
Le Bruit Du Frigo	Mano Negra	N
El Gato Lopez	Ska-P	Y

Table S2 – Group differences in BAT scores

Model fit: R^2=0.48; F(6, 55)=8.46; *p*<0.001

	β estimate	SE	*t*	*p* value	95% CI
Intercept	1.68	1.02	1.64	0.11	[-0.37, 3.72]
Group*	1.62	0.31	5.23	<0.001	[1.00, 2.25]
Age	-0.0086	0.0096	-0.89	0.38	[-0.028, 0.011]
Sex	-0.48	0.32	-1.48	0.14	[-1.12, 0.17]
Education	-0.064	0.066	-0.97	0.34	[-0.20, 0.068]
Handedness	-0.25	0.40	-0.61	0.54	[-1.06, 0.56]
MES*	0.11	0.24	4.51	<0.001	[0.059, 0.15]

Notes. Variable coding same as in Table 2 in the main text. * denotes significant predictor.

Table S3 – Language and motor abilities in unimpaired vs. impaired tappers

	Impaired tappers (n=13)	Unimpaired tappers (n=20)
MES	11.1 + 7.6	14.3 + 5.7
AST – L. side	0.5 + 0.1	0.7 + 0.3
NIHSS – R. side	1.2 + 1.2	0.5 + 0.9
QAB Overall	5.4 + 2.2	7.1 + 2.9
QAB SWC	9.0 + 1.2	9.1 + 2.2
QAB SC	5.0 + 3.7	6.7 + 3.7
QAB Word finding	3.4 + 2.4	6.2 + 3.2
QAB GC	4.1 + 3.6	7.2 + 3.4
QAB SMP	7.7 + 2.2	7.4 + 3.8
QAB Repetition	5.5 + 2.1	7.3 + 3.1
QAB Reading	4.1 + 3.8	6.5 + 3.5
QAB Dysarthria	8.5 + 2.4	9.2 + 1.6

Notes. Values are the mean + standard deviation. MES = Musical experience survey; AST = Apraxia Screen of TULIA; NIHSS = NIH Stroke Scale; QAB = Quick Aphasia Battery; SWC = Single word comprehension; SC = Sentence comprehension; GC = Grammatical construction; SMP = Speech motor programming.

Table S4 – Multiple linear regressions with motor variables

Model M1 fit: R^2=0.23; F(3, 30)=4.55; *p*=0.019

Model M2 fit: R^2=0.027; F(3, 30)=0.41; *p*=0.67

	β estimate	SE	*t*	*p* value	95% CI
Model M1 – tapping					
Intercept	21.99	13.45	1.64	0.11	[-5.47, 49.45]
AST* - L. side	39.50	18.49	2.14	0.041	[1.73, 77.26]
NIHSS – R. arm	-6.03	4.41	-1.37	0.18	[-15.03, 2.97]
Model M2 – BAT					
Intercept	0.86	0.72	1.20	0.24	[-0.61, 2.33]
AST – L. side	0.89	0.99	0.90	0.38	[-1.13, 2.91]
NIHSS – R. arm	0.043	0.24	0.18	0.86	[-0.44, 0.52]

Notes. AST=Apraxia Screen of TULIA; NIHSS=NIH Stroke Scale.

Table S5 – Multiple linear regressions with stroke variables

Model S1 fit: R^2=0.0079; F(3, 30)=0.12; *p*=0.89

Model S2 fit: R^2=0.0094; F(3, 30)=0.14; *p*=0.87

	β estimate	SE	*t*	*p* value	95% CI
Model S1 – tapping					
Intercept	39.19	8.20	4.78	<0.001	[22.44, 55.94]
Stroke Type	6.95	14.40	0.48	0.63	[-22.46, 36.37]
MPO	0.015	0.099	0.15	0.88	[-0.19, 0.22]
Model S2 – BAT					
Intercept	1.59	0.39	4.08	<0.001	[0.79, 2.38]
Stroke type	-0.067	0.68	-0.098	0.92	[-1.46, 1.33]
MPO	-0.0025	0.0047	-0.53	0.60	[-0.012, 0.0071]

Notes. Variable coding for binary variables: Stroke type (1=hemorrhagic, 0=ischemic). MPO=months post-onset.

Figure S1

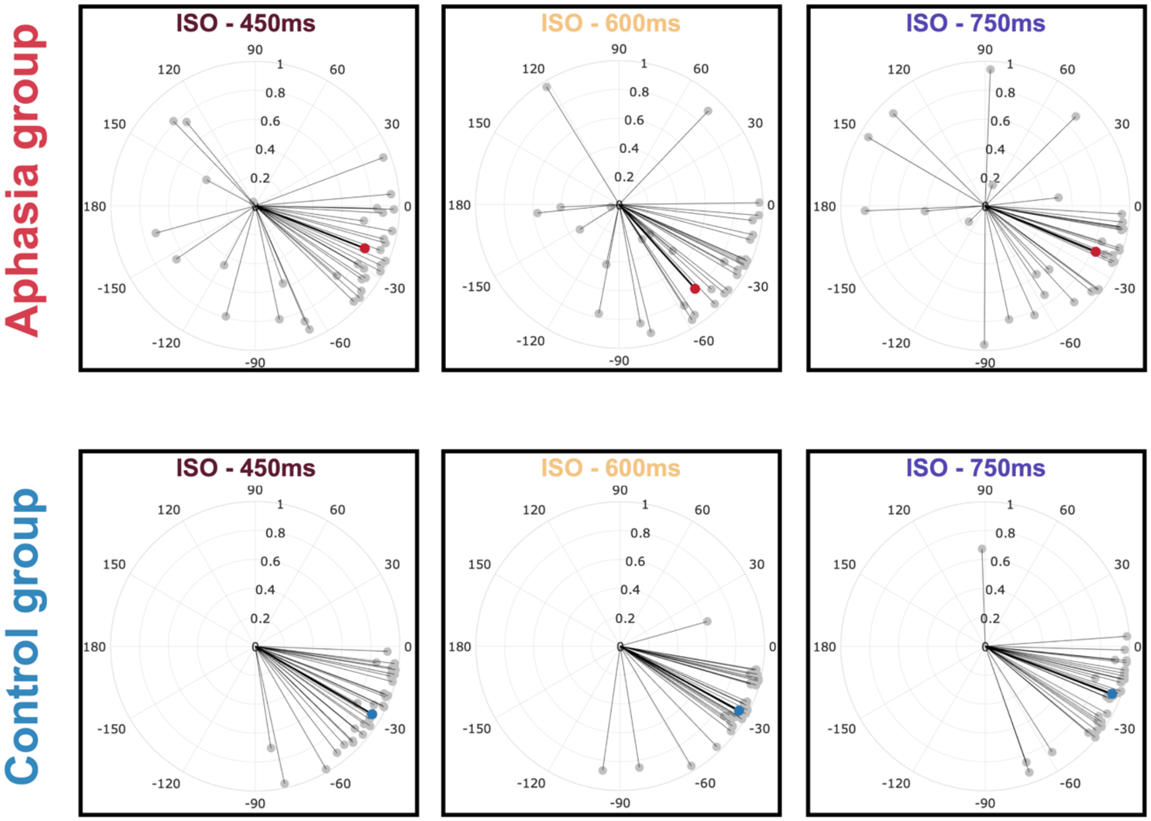

Figure S1. Individual-level participant tapping for each isochronous clip. Top row: aphasia group bottom row: control group. Each vector is a participant and colored vectors are the group mean. Data were analyzed using circular statistics.

Figure S2

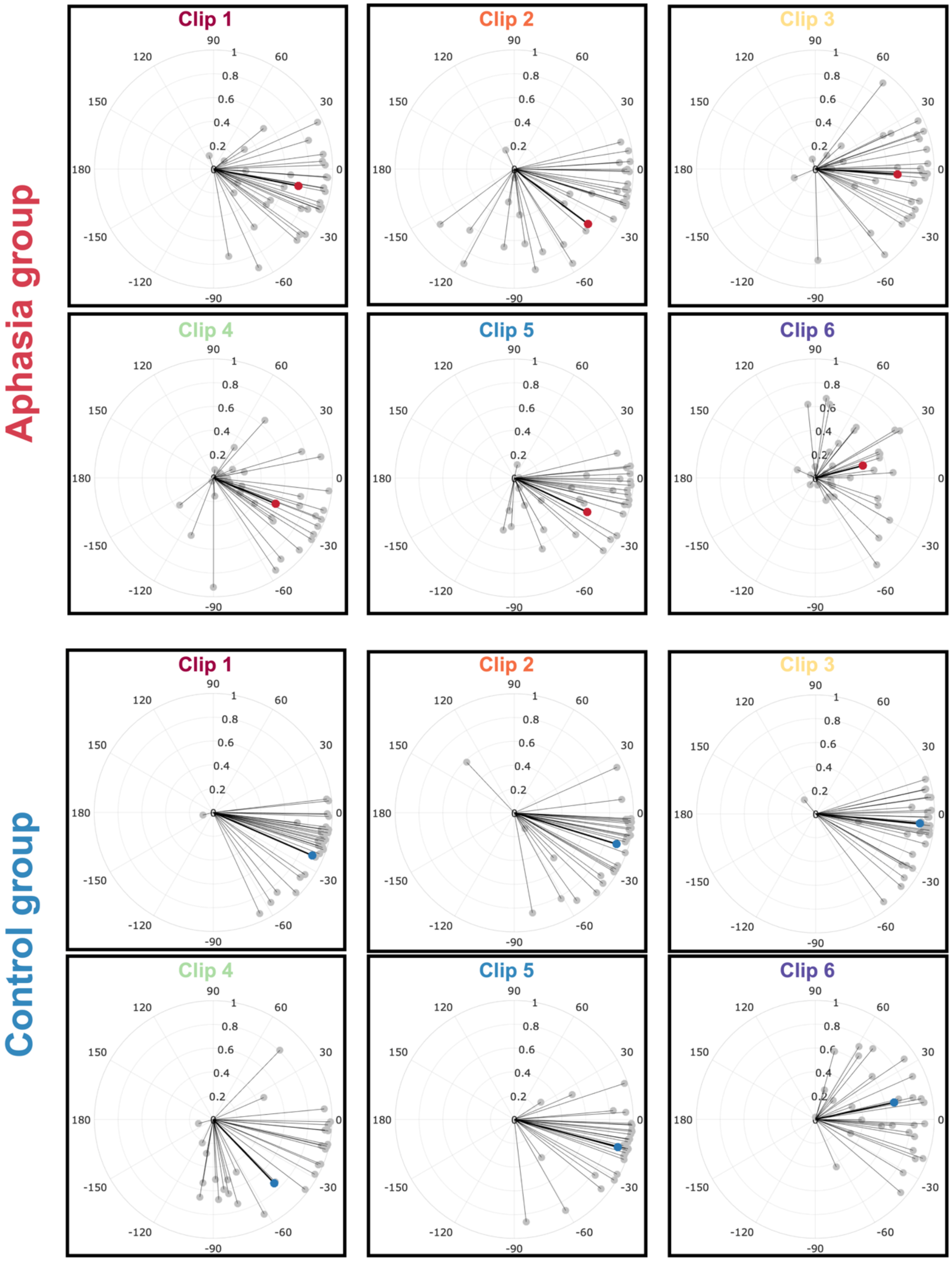

Figure S2. Individual participant tapping data for each music clip. See above for more information.

Figure S3

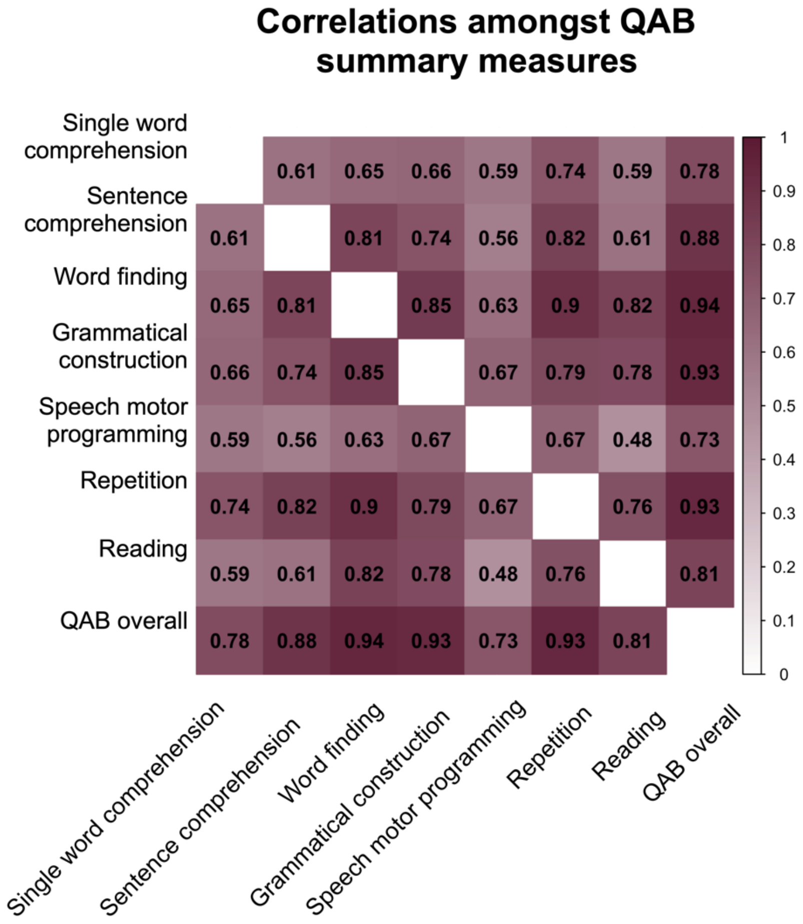

Figure S3. Correlation matrix for QAB summary measures. Color bar shows Pearson's *r* values.

Figure S4

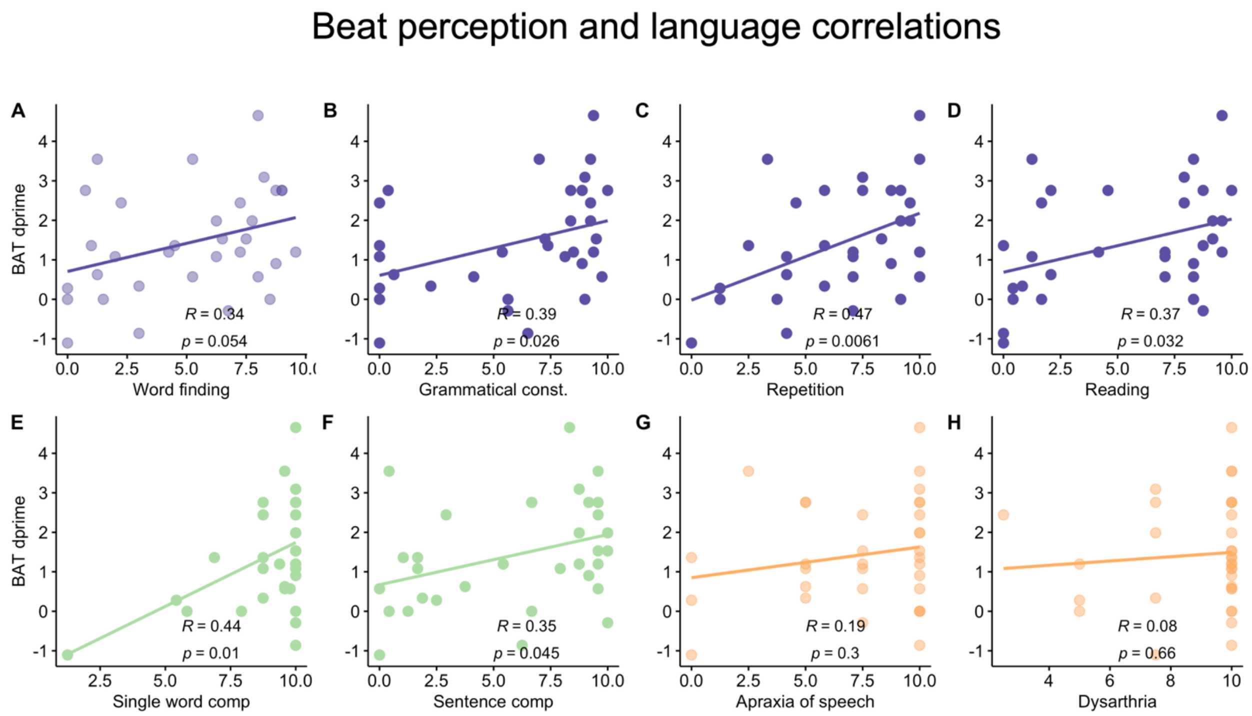

Figure S4. Scatter plots showing correlations between the BAT and each QAB summary measure. Purple=language production; green=language perception; orange=motor speech. Panels with lighter shaded dots indicate correlations did not reach statistical significance (uncorrected).

Figure S5

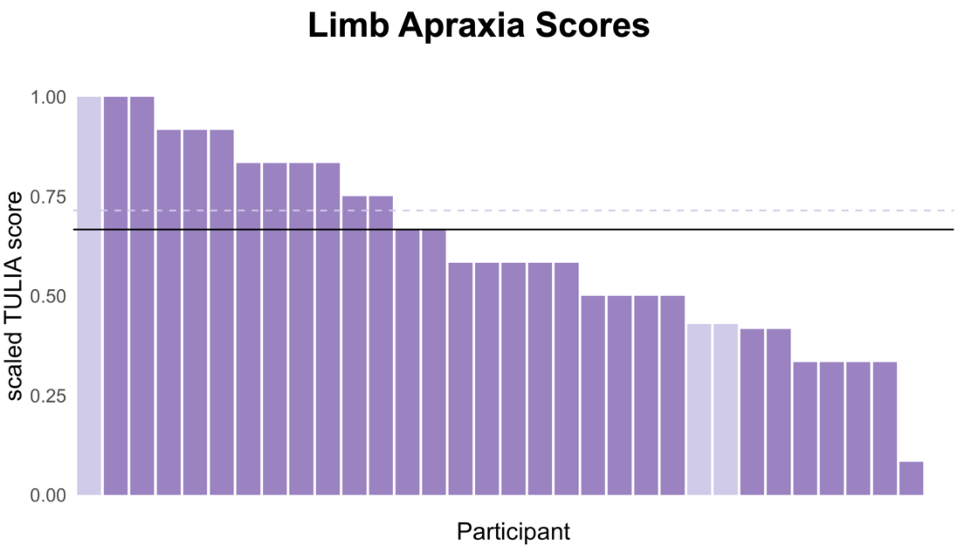

Figure S5. Scaled Apraxia Screen of TULIA (AST) scores. Scores are for the left side of the body (i.e., the side participants tapped with). The solid black line is the limb apraxia cut-off score for individuals who were scored on all 12 items (<9 indicative of limb apraxia); the dotted light purple line is the cut-off for individuals with significant comprehension deficits who were only scored on 7 items (<5 indicative of limb apraxia). Participants scored on only the 7 imitation items are shaded in light purple.
